# Matrix Riemann–Hilbert problems with jumps across Carleson contours

**DOI:** 10.1007/s00605-017-1019-0

**Published:** 2017-01-28

**Authors:** Jonatan Lenells

**Affiliations:** Department of Mathematics, KTH Royal Institute of Technology, 100 44 Stockholm, Sweden

**Keywords:** Matrix Riemann–Hilbert problem, Cauchy integral, Carleson contour, 35Q15, 30E25, 45E05

## Abstract

We develop a theory of $n \times n$-matrix Riemann–Hilbert problems for a class of jump contours and jump matrices of low regularity. Our basic assumption is that the contour $\Gamma$ is a finite union of simple closed Carleson curves in the Riemann sphere. In particular, unbounded contours with cusps, corners, and nontransversal intersections are allowed. We introduce a notion of $L^p$-Riemann–Hilbert problem and establish basic uniqueness results and Fredholm properties. We also investigate the implications of Fredholmness for the unique solvability and prove a theorem on contour deformation.

## Introduction

A Riemann–Hilbert (RH) problem consists of finding a sectionally analytic function with prescribed jumps across some given contour in the complex plane. In its simplest formulation, the problem involves a smooth simple closed contour $\Gamma$ dividing the complex plane into an interior domain $D_+$ and an exterior domain $D_-$, as well as a smooth ‘jump matrix’ *v*(*z*) defined for $z \in \Gamma$. The problem consists of finding an $n\times n$-matrix-valued function *m*(*z*) which is analytic in $D_+ \cup D_-$ and whose boundary values $m_+$ and $m_-$ from the left and right sides of $\Gamma$ exist, are continuous, and satisfy the jump condition $m_+ = m_- v$ on $\Gamma$. Uniqueness is ensured by requiring that *m* approaches the identity matrix at infinity.

The theory of scalar RH problems is well-developed in the classical set-up in the complex plane [26] as well as for problems on Riemann surfaces [28, 30]. Constructive existence and uniqueness results are available, at least within classes of Hölder continuous functions [1, 26, 30]. We refer to the monograph [22] for more recent developments and further references in the case of less regular solutions.

The theory of matrix RH problems is substantially more complicated than the scalar theory. Only very special classes of problems (such as problems with a rational jump matrix, see Chapter I of [6]) can be solved explicitly. Uniqueness can often be established by means of Liouville’s theorem, but existence results are rare and usually rely on the presence of some special symmetry, see [1, 8].

Matrix RH problems are essential in the analysis of integrable systems, orthogonal polynomials, and random matrices. The RH approach is particularly powerful when it comes to determining asymptotics. Indeed, the asymptotic behavior of solutions of many RH problems can be efficiently determined by means of the nonlinear steepest descent method introduced by Deift and Zhou [10], building on earlier work of Its [19] and Manakov [25]. This method and generalizations thereof have been instrumental in several recent advances in random matrix theory and in the analysis of large-time asymptotics of solutions of integrable PDE’s [8, 9, 15, 16, 20, 21].

The classical formulation of a RH problem, which involves a piecewise smooth contour $\Gamma$ and a smooth (or at least Hölder continuous) jump matrix *v*, is sufficient for many applications. However, in order to obtain a more convenient setting for the application of functional analytic techniques, it is essential to extend the formulation of a RH problem to the $L^p$-setting [5, 24]. Deift and Zhou and others [8, 11, 12, 16, 29] have extended the definition of a RH problem to the case where the jump matrix *v* and its inverse $v^{-1}$ belong to appropriate Lebesgue spaces, and the contour $\Gamma$ is a finite union of closed simple smooth curves in the Riemann sphere with a finite number of transversal intersection points. In particular, the relationship between the unique solvability of a RH-problem and the Fredholmness of a certain associated singular operator was explained in [29].

Our goal in this paper is to lay the foundation for a theory of matrix RH problems for a class of possibly unbounded jump contours of very low regularity. Our basic assumption is that the contour $\Gamma$ is a finite union of closed Carleson curves in the Riemann sphere. The contours are allowed to pass through infinity and to have cusps, corners, and nontransversal intersections. We introduce a notion of $L^p$-Riemann–Hilbert problem for this class of contours and establish basic uniqueness results and Fredholm properties. We also investigate the implications of Fredholmness for the unique solvability and prove a theorem on contour deformation. We mainly develop those parts of the theory which seem most relevant for applications to integrable equations. For example, at several places in Sect. 5 we assume that the jump matrix has unit determinant and we do not consider possible generalizations of partial indices.

The matrix RH problems considered here are different from the vector RH problems studied, for example, in [4] and [24]. However, the Fredholm theories of these two problems are closely related, so in this regard our main contribution is to extend results known for bounded curves to unbounded curves. Such an extension is important for applications to integrable equations where most contours naturally pass through infinity.

The formulation of a successful theory of RH problems is intricately linked to the boundedness of the Cauchy singular operator $\mathcal {S}_\Gamma$ defined in Eq. (2.3) below. Indeed, this operator is the key ingredient in the Sokhotski-Plemelj formulas for the boundary values of an analytic function. Since it has been proved in recent years that $\mathcal {S}_\Gamma$ is a bounded operator on $L^p(\Gamma )$ if and only if $\Gamma$ is Carleson (cf. [4]), it is natural to expect that the class of Carleson contours is the most general class of contours for which a clean RH theory exists. This is the reason we choose to consider Carleson jump contours.

**Fig. 1:**
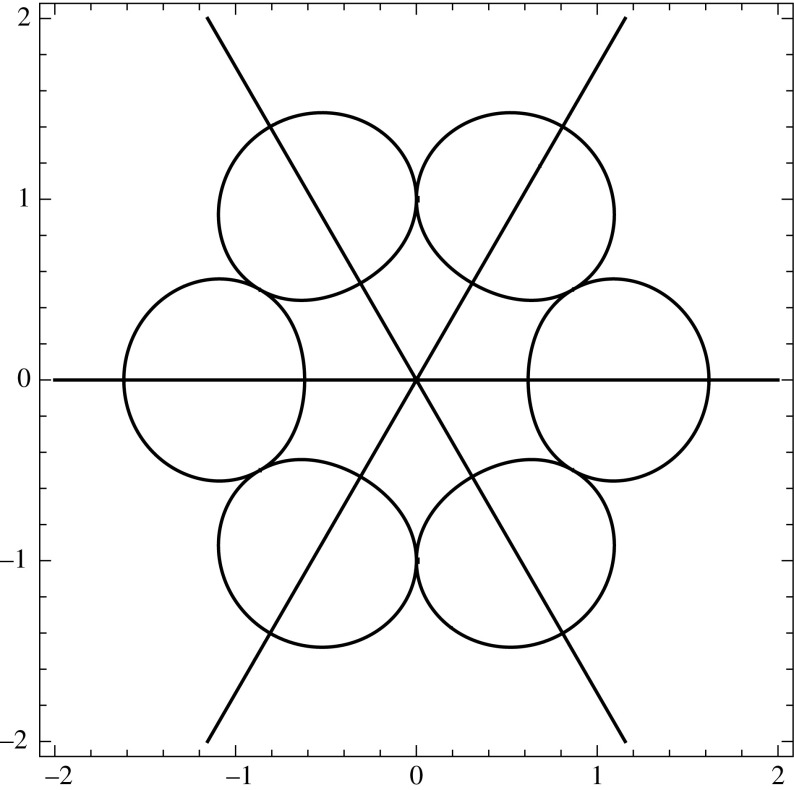
A jump contour with nontransversal intersections that arises in the analysis of the Degasperis–Procesi equation on the half-line

We emphasize that RH problems with contours involving nontransversal intersections are important in applications to integrable evolution equations. For example, the analysis of the Degasperis-Procesi equation on the half-line naturally leads to a RH problem with the jump contour displayed in Fig. 1, see [23]. The results of the present paper can be used to rigorously derive the long-time asymptotics of the solutions of this equation via the nonlinear steepest descent method [3].

An additional reason for writing this paper is to make accessible detailed and rigorous proofs of several basic results on matrix RH problem. Many of these results are well-known to the experts (at least if the contour $\Gamma$ is sufficiently well-behaved), but their proofs are scattered or absent in the literature. It turns out that the basic results can be proved in the more general setting of Carleson jump contours with little extra effort.

In Sect. 2, we summarize several properties of Smirnoff classes and Cauchy integrals over rectifiable Jordan curves. In Sect. 3, we introduce the notion of a Carleson jump contour as well as a number of function spaces which turn out to be convenient when dealing with contours passing through infinity. In Sect. 4, we establish several properties of Cauchy integrals over general Carleson jump contours. In Sect. 5, we introduce a notion of $L^p$-Riemann–Hilbert problem for a general Carleson jump contour and develop the basics of a theory for these problems.

## Preliminaries

A subset $\Gamma \subset \mathbb {C}$ is an *arc* if it is homeomorphic to a connected subset *I* of the real line which contains at least two distinct points. If $\varphi :I \rightarrow \Gamma$ is a homeomorphism onto an arc and $(a,b) \subset I$ is the interior of *I* with $a \in \mathbb {R}\cup \{-\infty \}$ and $b \in \mathbb {R}\cup \{\infty \}$, then $\lim _{t \rightarrow a^+} \varphi (t)$ and $\lim _{t \rightarrow b^-} \varphi (t)$ are referred to as *endpoints* of $\Gamma$ whenever the limits exist and are finite. An arc may have two, one, or no endpoints. An arc that does not contain its endpoints is an *open* arc. If $I = [a,b]$ is a closed interval, the *length* $|\Gamma |$ of $\Gamma$ is defined by

$$\begin{aligned} |\Gamma | = \sup \sum _{i=1}^n |\varphi (t_i) - \varphi (t_{i-1})| \end{aligned}$$

where the supremum is over all partitions $a = t_0< t_1< \cdots < t_n = b$ of [*a*, *b*]. If *I* is not closed, the length of $\Gamma$ is defined as the supremum of $|\varphi ([a,b])|$ as [*a*, *b*] ranges over all closed subintervals of *I*. The arc $\Gamma$ is *rectifiable* if its length is finite. A subset $\Gamma \subset \mathbb {C}$ is a *composed curve* if it is connected and may be represented as the union of finitely many arcs each pair of which have at most endpoints in common. A composed curve is *oriented* if it can be represented as the union of finitely many oriented arcs each pair of which have at most endpoints in common. A subset $\Gamma \subset \mathbb {C}$ is a *Jordan curve* if it is homeomorphic to the unit circle $S^1$.

Let $\Gamma \subset \mathbb {C}$ be a composed curve. If $z \in \mathbb {C}$, $r \in (0, \infty )$, and *D*(*z*, *r*) denotes the open disk of radius *r* centered at *z*, then $\Gamma \cap D(z, r)$ is an at most countable union of arcs. If all of these arcs are rectifiable and the sum of their lengths is finite, we say that $\Gamma \cap D(z, r)$ is rectifiable. $\Gamma$ is *locally rectifiable* if $\Gamma \cap D(z, r)$ is rectifiable for every $z \in \Gamma$ and every $r \in (0,\infty )$. A composed curve $\Gamma$ is locally rectifiable if and only if $\Gamma \cap D(0, r)$ is rectifiable for every $r \in (0,\infty )$.

### Carleson curves

Let $\Gamma \subset \mathbb {C}$ be a locally rectifiable composed curve. We equip $\Gamma$ with Lebesgue length measure and denote the measure of a measurable subset $\gamma \subset \Gamma$ by $|\gamma |$; see e.g. Chapter 1 of [27] for a detailed definition. We say that $\Gamma$ is *Carleson* (or a *Carleson curve*) if

2.1 $$\begin{aligned} \sup _{z \in \Gamma } \sup _{r > 0} \frac{|\Gamma \cap D(z, r)|}{r} < \infty . \end{aligned}$$

The condition (2.1) is equivalent to the condition

2.2 $$\begin{aligned} \sup _{z \in \mathbb {C}} \sup _{r > 0} \frac{|\Gamma \cap D(z, r)|}{r} < \infty . \end{aligned}$$

Moreover, $\Gamma$ is Carleson if and only if each of its finite number of arcs is Carleson. We refer to Chapter 1 of [4] for more information on Carleson curves.

### Cauchy singular operator

Let $\Gamma$ be a composed locally rectifiable curve. Let $C_0^\infty (\Gamma )$ denote the set of all restrictions of smooth functions $f:\mathbb {R}^2 \rightarrow \mathbb {C}$ of compact support to $\Gamma$. A measurable function $w:\Gamma \rightarrow [0,\infty ]$ is a *weight* on $\Gamma$ if the preimage $w^{-1}(\{0,\infty \})$ has measure zero. The weighted Lebesgue space $L^p(\Gamma , w)$, $p \in [1, \infty )$, is defined as the space of measurable functions such that

$$\begin{aligned} \Vert f\Vert _{L^p(\Gamma , w)} := \bigg (\int _\Gamma |f(z)|^p w(z)^p |dz|\bigg )^{1/p} < \infty . \end{aligned}$$

Equipped with the norm $\Vert \cdot \Vert _{L^p(\Gamma , w)}$, $L^p(\Gamma , w)$ is a Banach space.

Given a function *h* defined on $\Gamma$, we define $(\mathcal {S}_\Gamma h)(z)$ for $z \in \Gamma$ by

2.3 $$\begin{aligned} (\mathcal {S}_\Gamma h)(z) = \lim _{\epsilon \rightarrow 0} \frac{1}{\pi i} \int _{\Gamma {\setminus } D(z, \epsilon )} \frac{h(z')}{z' - z} dz', \end{aligned}$$

whenever the limit exists. If $h \in C_0^\infty (\Gamma )$, then $(\mathcal {S}_\Gamma h)(z)$ exists for almost all $z \in \Gamma$ (see Theorem 4.14 of [4]). If *w* is a weight on $\Gamma$, we say that the Cauchy singular operator $\mathcal {S}_\Gamma$ *generates a bounded operator on* $L^p(\Gamma , w)$ if $C_0^\infty (\Gamma )$ is dense in $L^p(\Gamma , w)$ and

$$\begin{aligned} \Vert \mathcal {S}_\Gamma f\Vert _{L^p(\Gamma , w)} < M \Vert f\Vert _{L^p(\Gamma ,w)} \quad \text {for all} \quad f \in C_0^\infty (\Gamma ) \end{aligned}$$

with some constant $M > 0$ independent of *f*; in that case there exists a unique bounded operator $\tilde{\mathcal {S}}_\Gamma$ on $L^p(\Gamma , w)$ such that $\tilde{\mathcal {S}}_\Gamma f = \mathcal {S}_\Gamma f$ for all $f \in C_0^\infty (\Gamma )$.

It was realized in the early eighties that the Carleson condition is the essential condition for ascertaining boundedness of $\mathcal {S}_\Gamma$ in $L^p$-spaces [7]. More precisely, if $1< p < \infty$ and $\Gamma$ is a composed locally rectifiable curve, then $\mathcal {S}_\Gamma$ generates a bounded operator on $L^p(\Gamma )$ if and only if $\Gamma$ is Carleson. In dealing with unbounded contours, we will need a more general version of this result valid for weighted $L^p$-spaces.

Let $p \in (1, \infty )$. We define $A_p(\Gamma )$ as the set of weights $w \in L_{\text {loc}}^p(\Gamma )$ such that $1/w \in L_{\text {loc}}^q(\Gamma )$ and

2.4 $$\begin{aligned} \sup _{z \in \Gamma } \sup _{r >0} \bigg (\frac{1}{r} \int _{\Gamma \cap D(z, r)} w(z')^p |dz'|\bigg )^{1/p}\bigg (\frac{1}{r} \int _{\Gamma \cap D(z, r)} w(z')^{-q} |dz'|\bigg )^{1/q} < \infty , \end{aligned}$$

where $q \in (1,\infty )$ is defined by $1/p + 1/q = 1$. Elements in $\cup _{1<p<\infty } A_p(\Gamma )$ are referred to as *Muckenhoupt weights* on $\Gamma$. If $\Gamma$ is Carleson, then constant weights belong to $A_p(\Gamma )$. If $A_p(\Gamma )$ is nonempty, then $\Gamma$ is Carleson.

#### Theorem 2.1

Let $1< p < \infty$ and let $\Gamma$ be a composed locally rectifiable curve. Let *w* be a weight on $\Gamma$. Then $\mathcal {S}_\Gamma$ generates a bounded operator $\tilde{\mathcal {S}}_\Gamma$ on $L^p(\Gamma , w)$ if and only if $\Gamma$ is Carleson and $w \in A_p(\Gamma )$. Moreover, if $f \in L^p(\Gamma , w)$ and $\mathcal {S}_\Gamma$ generates a bounded operator on $L^p(\Gamma , w)$, then the limit in (2.3) exists and $(\mathcal {S}_\Gamma f)(z) = (\tilde{\mathcal {S}}_\Gamma f)(z)$ for a.e. $z \in \Gamma$.

#### Proof

See Theorem 4.15 and Remark 5.23 of [4]. $\square$

### Smirnoff classes

Let $\Gamma \subset \mathbb {C}$ be a rectifiable Jordan curve oriented counterclockwise. Let $\hat{\mathbb {C}} = \mathbb {C}\cup \infty$ denote the Riemann sphere and let $D_+$ and $D_-$ be the two components of $\hat{\mathbb {C}} {\setminus } \Gamma$. Assuming that $\infty \in D_-$, we refer to $D_+$ and $D_-$ as the interior and exterior components respectively. Let $1 \le p < \infty$. A function *f* analytic in $D_+$ belongs to the *Smirnoff class* $E^p(D_+)$ if there exists a sequence of rectifiable Jordan curves $\{C_n\}_1^\infty$ in $D_+$, tending to the boundary in the sense that $C_n$ eventually surrounds each compact subdomain of $D_+$, such that

2.5 $$\begin{aligned} \sup _{n \ge 1} \int _{C_n} |f(z)|^p |dz| < \infty . \end{aligned}$$

A function *f* analytic in $D_-$ is said to be of class $E^p(D_-)$ if there exists a sequence of rectifiable Jordan curves $\{C_n\}_1^\infty$ in $D_-$, tending to the boundary $\Gamma$ in the sense that every compact subset of $D_-$ eventually lies outside $\Gamma _n$, such that (2.5) holds. We let $\dot{E}^p(D_-)$ denote the subspace of $E^p(D_-)$ consisting of all functions $f \in E^p(D_-)$ that vanish at infinity.

### Basic results on Cauchy integrals

Given a locally rectifiable composed contour $\Gamma \subset \mathbb {C}$ and a measurable function *h* defined on $\Gamma$, we define the Cauchy integral $(\mathcal {C}h)(z)$ for $z \in \mathbb {C}{\setminus }\Gamma$ by

2.6 $$\begin{aligned} (\mathcal {C}h)(z) = \frac{1}{2\pi i} \int _\Gamma \frac{h(z')dz'}{z' - z}, \end{aligned}$$

whenever the integral converges. To avoid confusion, we will sometimes indicate the dependence of $\mathcal {C}$ on $\Gamma$ by writing $\mathcal {C}_\Gamma$ for $\mathcal {C}$.

In the next two propositions, we collect a number of properties of the Cauchy integral and its relation to the Smirnoff classes; we refer to Chapter 10 of [14] and Chapter 6 of [4] for proofs. Given a Jordan curve $\Gamma {\subset }\mathbb {C}$, we let $D_+$ and $D_-$ denote the interior and exterior components of $\hat{\mathbb {C}} {\setminus } \Gamma$.

#### Theorem 2.2

Let $\mathcal {C}$ denote the Cauchy integral operator defined in (2.6).

- Let $1 \le p < \infty$. Suppose $\Gamma \subset \mathbb {C}$ is a rectifiable Jordan curve. If $f \in E^p(D_+)$, then the nontangential limits of *f*(*z*) as *z* approaches the boundary exist a.e. on $\Gamma$; if $f_+(z)$ denotes the boundary function, then $f_+ \in L^p(\Gamma )$ and
  2.7 $$\begin{aligned} (\mathcal {C} f_+)(z) = {\left\{ \begin{array}{ll} f(z), &{}\quad z \in D_+, \\ 0, &{}\quad z \in D_-. \end{array}\right. } \end{aligned}$$
  If $f \in E^p(D_-)$, then the nontangential limits of *f*(*z*) as *z* approaches the boundary exist a.e. on $\Gamma$. If $f_-(z)$ denotes the boundary function, then $f_- \in L^p(\Gamma )$ and
  2.8 $$\begin{aligned} (\mathcal {C} f_-)(z) = {\left\{ \begin{array}{ll} f(\infty ), &{}\quad z \in D_+, \\ f(\infty ) - f(z), &{}\quad z \in D_-. \end{array}\right. } \end{aligned}$$
- Let $1< p < \infty$. Suppose $\Gamma$ is a Carleson Jordan curve. Then the Cauchy singular operator $\mathcal {S}_\Gamma :L^p(\Gamma ) \rightarrow L^p(\Gamma )$ defined in (2.3) satisfies $\mathcal {S}_\Gamma ^2 = I$. Moreover, if $h \in L^p(\Gamma )$, then
  $$\begin{aligned} (\mathcal {C}h)|_{D_+} \in E^p(D_+), \qquad (\mathcal {C}h)|_{D_-} \in \dot{E}^p(D_-). \end{aligned}$$

Theorem 2.2 implies that if $\Gamma$ is a Carleson Jordan curve and $h \in L^p(\Gamma )$ for some $1< p < \infty$, then the left and right nontangential boundary values of $\mathcal {C}h$, which we denote by $\mathcal {C}_+ h$ and $\mathcal {C}_- h$, lie in $L^p(\Gamma )$. This allows us to view $\mathcal {C}_\pm$ as linear operators $\mathcal {C}_\pm :h \mapsto \mathcal {C}_\pm h$ on $L^p(\Gamma )$.

#### Theorem 2.3

Let $1< p < \infty$ and let $\Gamma \subset \mathbb {C}$ be a Carleson Jordan curve. Then $\mathcal {C}_\pm$ are bounded operators on $L^p(\Gamma )$ with the following properties:

- The Sokhotski-Plemelj formulas
  $$\begin{aligned} \mathcal {C}_+ = \frac{1}{2}(I + \mathcal {S}_\Gamma ), \qquad \mathcal {C}_- = \frac{1}{2}(-I + \mathcal {S}_\Gamma ), \end{aligned}$$
  are valid.
- $\mathcal {C}_\pm$ are complementary projections on $L^p(\Gamma )$ in the sense that
  $$\begin{aligned} L^p(\Gamma ) = \mathcal {C}_+L^p(\Gamma ) \oplus \mathcal {C}_-L^p(\Gamma ) \end{aligned}$$
  and
  $$\begin{aligned} \mathcal {C}_+ - \mathcal {C}_- = \mathbb {I}, \qquad \mathcal {C}_+^2 = \mathcal {C}_+, \qquad \mathcal {C}_-^2 = -\mathcal {C}_-, \qquad \mathcal {C}_+\mathcal {C}_- = \mathcal {C}_-\mathcal {C}_+ = 0. \end{aligned}$$
- If $$h = \mathcal {C}_+h - \mathcal {C}_-h \in L^p(\Gamma )$$, then
  $$\begin{aligned} (\mathcal {C}h)|_{D_+} = (\mathcal {C}\mathcal {C}_+h)|_{D_+} \in E^p(D_+), \qquad (\mathcal {C}h)|_{D_-} = -(\mathcal {C}\mathcal {C}_-h)|_{D_-} \in \dot{E}^p(D_-). \end{aligned}$$
- The map $h \mapsto (\mathcal {C}h)|_{D_+}$ is a bijection $\mathcal {C}_+L^p(\Gamma ) \rightarrow E^p(D_+)$ with inverse $f \mapsto f_+$.
- The map $h \mapsto (\mathcal {C}h)|_{D_-}$ is a bijection $\mathcal {C}_-L^p(\Gamma ) \rightarrow \dot{E}^p(D_-)$ with inverse $f \mapsto -f_-$.

## Carleson jump contours

RH problems are conveniently formulated on the Riemann sphere $\hat{\mathbb {C}} = \mathbb {C}\cup \infty$. In order to allow for jump contours passing through infinity, we introduce a class of curves $\mathcal {J}$, which in addition to the rectifiable Jordan curves considered in the previous section also includes unbounded contours. Recall that $\Gamma \subset \mathbb {C}$ is referred to as a Carleson curve if $\Gamma$ is a locally rectifiable composed curve satisfying (2.1). We extend this notion to the Riemann sphere by calling a subset $\Gamma \subset \hat{\mathbb {C}}$ a Carleson curve if and only if $\Gamma$ is connected and $\Gamma \cap \mathbb {C}$ is a Carleson curve.

### The class $\mathcal {J}$

Let $\mathcal {J}$ denote the collection of all subsets $\Gamma$ of the Riemann sphere $\hat{\mathbb {C}}$ such that $\Gamma$ is homeomorphic to the unit circle and $\Gamma$ is a Carleson curve. If $\infty \notin \Gamma$, then $\Gamma \in \mathcal {J}$ if and only if $\Gamma \subset \mathbb {C}$ is a Carleson Jordan curve. However, $\mathcal {J}$ also includes curves passing through infinity. In fact, the next proposition shows that $\mathcal {J}$ is invariant under the action of the group of linear fractional transformations. This shows that $\mathcal {J}$ is a natural extension of the family of Carleson Jordan curves in that it puts $\infty$ on an equal footing with the other points in the Riemann sphere.

#### Proposition 3.1

The family of all Carleson curves in $\hat{\mathbb {C}}$ is invariant under the action of the group of linear fractional transformations. In other words, if $\psi : \hat{\mathbb {C}} \rightarrow \hat{\mathbb {C}}$ is given by

3.1 $$\begin{aligned} \psi (z) = \frac{az + b}{cz + d}, \end{aligned}$$

for some constants $a,b,c,d \in \mathbb {C}$ with $ad - bc \ne 0$, then $\Gamma \subset \hat{\mathbb {C}}$ is a Carleson curve if and only if $\psi (\Gamma ) \subset \hat{\mathbb {C}}$ is a Carleson curve.

#### Proof

See “Appendix 1”. $$\square $$

#### Remark 3.2

If $\gamma :S^1 \rightarrow \hat{\mathbb {C}}$ is an injective $C^1$ map such that $|\gamma '(s)| \ne 0$ for all $s \in S^1$, then $\Gamma := \gamma (S^1)$ belongs to $\mathcal {J}$. Indeed, being a continuous bijection from a compact space onto a Hausdorff space, $\gamma$ is a homeomorphism $S^1 \rightarrow \Gamma$. In view of Proposition 3.1, we may assume that $\infty \notin \Gamma$. Then, since $S^1$ is compact, we may cover $S^1$ with a finite number of open sets $\{U_j\}_1^n$ such that the restriction of $\gamma$ to each $U_j$ is a $C^1$ graph; Proposition 1.1 of [4] now implies that $\Gamma$ is Carleson.

#### Remark 3.3

The Carleson condition is essential in Proposition 3.1. In fact, the family of composed locally rectifiable (but not necessarily Carleson) curves is *not* invariant under the action of the group of linear fractional transformations. Indeed, let $\Gamma = \{te^{-it^2} | 1< t < \infty \}$ and $\psi (z) = z^{-1}$. Then $\Gamma$ is locally rectifiable, but $\psi (\Gamma )$ is not locally rectifiable because $\psi (\Gamma ) = \{t^{-1}e^{it^2} | 1< t < \infty \} \subset D(0,1)$ has infinite length:

$$\begin{aligned} \int _1^\infty \bigg |\frac{d}{dt} t^{-1}e^{it^2} \bigg | dt = \int _1^\infty \sqrt{4 + \frac{1}{t^4}} dt = \infty . \end{aligned}$$

This example does not contradict Proposition 3.1. Indeed, the estimate

$$\begin{aligned} \frac{|\Gamma \cap D(0, r)|}{r} = \frac{1}{r}\int _1^r \bigg |\frac{d}{dt} te^{-it^2}\bigg | dt = \frac{1}{r}\int _1^r \sqrt{1 + 4t^4} dt > \frac{1}{r} \int _1^r 2t^2 dt = \frac{2(r^3 - 1)}{3r} \end{aligned}$$

implies that $\frac{|\Gamma \cap D(0, r)|}{r}$ is unbounded as $r \rightarrow \infty$; hence $\Gamma$ is not Carleson.

### Carleson jump contours

We call a connected subset $\Gamma \subset \hat{\mathbb {C}}$ of the Riemann sphere a *Carleson jump contour* if it has the following properties:

- $\Gamma \cap \mathbb {C}$ is an oriented composed curve.
- $\hat{\mathbb {C}}{\setminus }\Gamma$ is the union of two disjoint open sets $D_+$ and $D_-$ each of which has a finite number of simply connected components in $\hat{\mathbb {C}}$.
- $\Gamma$ is the positively oriented boundary of $D_+$ and the negatively oriented boundary of $D_-$, i.e. $\Gamma = \partial D_+ = -\partial D_-$.
- If $\{D_j^+\}_1^n$ and $\{D_j^-\}_1^m$ are the components of $D_+$ and $D_-$, then $\partial D_j^+ \in \mathcal {J}$ for $j = 1, \ldots , n$, and $\partial D_j^- \in \mathcal {J}$ for $j = 1, \ldots , m$.

**Fig. 2:**
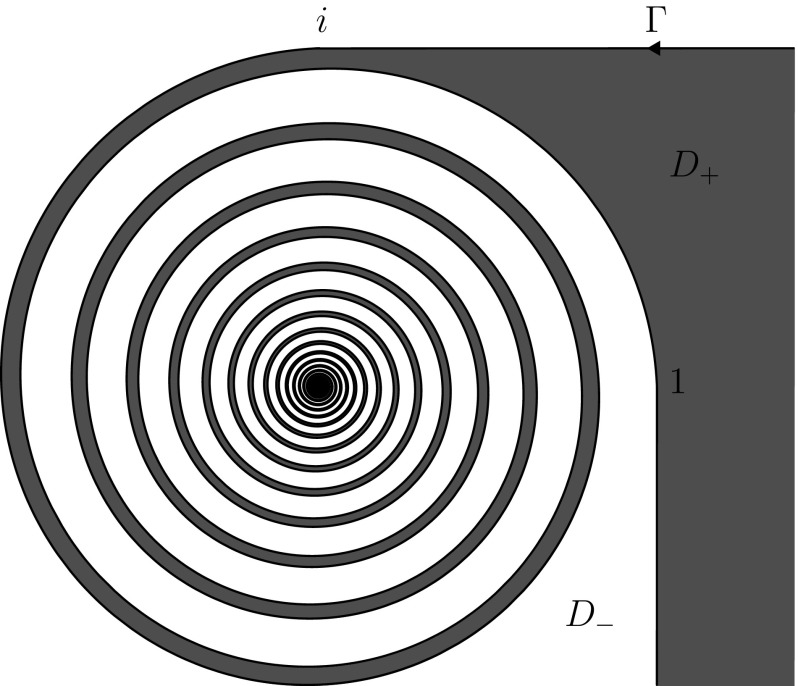
The logarithmic spiral defined in (3.2) is an example of a Carleson jump contour (see [4, page 12] for an analogous, but bounded, example)

#### Example 3.4

The curve $\Gamma \subset \mathbb {C}$ defined by (see Fig. 2)

**Fig. 3:**
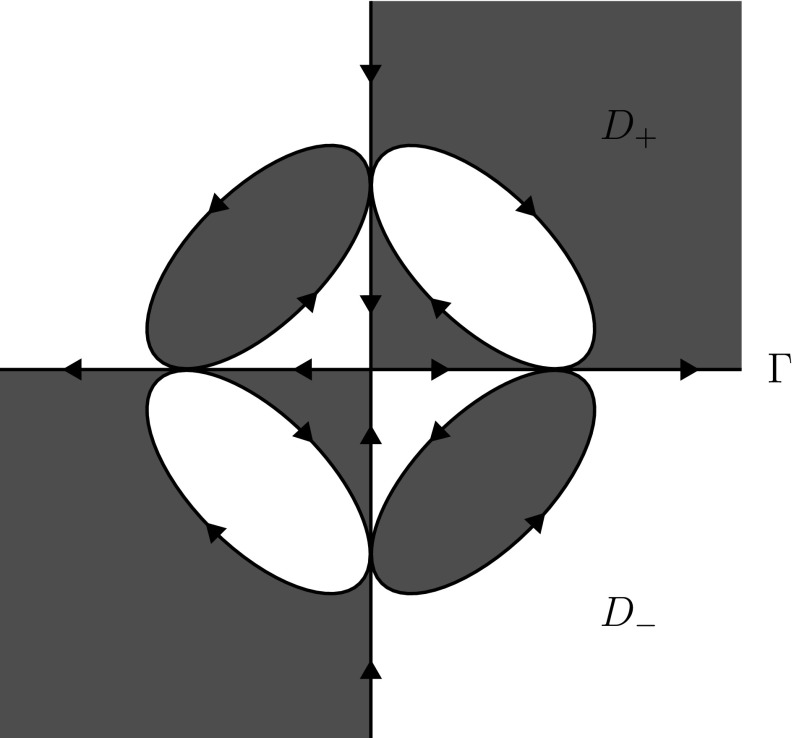
An example of a Carleson jump contour

**Fig. 4:**
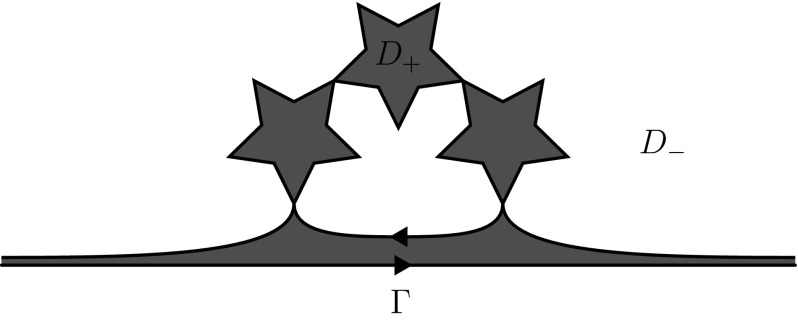
An example of a Carleson jump contour

3.2 $$\begin{aligned} \Gamma =&\; \{1 - ir \, | \, 0 \le r< \infty \} \cup \{i + r \, | \, 0 \le r< \infty \} \cup \{\infty \} \nonumber \\&\; \cup \{re^{-25 i \ln r} \, | \, 0< r < 1\} \cup \{0\} \end{aligned}$$

is a Carleson jump contour. Indeed, using that any logarithmic spiral $\{re^{-i\delta \ln r} \, | \, 0< r < 1\}$ for $\delta \in \mathbb {R}$ is a Carleson arc (see Example 1.6 of [4]), it is straightforward to show that $\Gamma$ is a Carleson Jordan curve. Other examples of Carleson jump contours are displayed in Figs. 3 and 4.

Proposition 3.1 implies the following result.

#### Proposition 3.5

The family of Carleson jump contours is invariant under the action of the group of linear fractional transformations. In other words, if $\psi : \hat{\mathbb {C}} \rightarrow \hat{\mathbb {C}}$ is given by (3.1) for some constants $a,b,c,d \in \mathbb {C}$ with $ad - bc \ne 0$, then $\Gamma$ is a Carleson jump contour if and only if $\psi (\Gamma )$ is a Carleson jump contour.

Our goal is to establish generalizations of Theorems 2.2 and 2.3 which are valid in the case of a general Carleson jump contour $\Gamma$. These generalizations will be stated and proved in Sect.  4; in the remainder of this section, we introduce a number of function spaces which will be needed for the formulation of these theorems.

### Generalized Smirnoff classes

In Sect. 2.3, we defined the Smirnoff class $E^p(D)$ for $D = D_+$ and $D = D_-$, where $D_+$ and $D_-$ are the domains interior and exterior to a rectifiable Jordan curve, respectively. We now extend this definition to allow for situations where *D* is an arbitrary finite disjoint union of domains bounded by curves in $\mathcal {J}$.

Let $1 \le p < \infty$. If *D* is a subset of $\hat{\mathbb {C}}$ bounded by a curve in $\mathcal {J}$ which passes through infinity, we define $E^p(D)$ as the set of functions *f* analytic in *D* for which $f \circ \varphi ^{-1} \in E^p(\varphi (D))$, where

3.3 $$\begin{aligned} \varphi (z) = \frac{1}{z - z_0} \end{aligned}$$

and $z_0$ is any point in $\mathbb {C}{\setminus }\Gamma$. It is easy to see that $E^p(D)$ does not depend on the choice of $z_0$. If *D* is a subset of $\hat{\mathbb {C}}$ bounded by a curve in $\mathcal {J}$, we define $\dot{E}^p(D)$ as the subspace of $E^p(D)$ consisting of all functions $f \in E^p(D)$ such that $z f(z) \in E^p(D)$. If *D* is bounded, then $\dot{E}^p(D) = E^p(D)$. If $\infty \in D$, then $\dot{E}^p(D)$ consists of the functions in $E^p(D)$ that vanish at infinity, so that the present definition of $\dot{E}^p(D)$ is consistent with the definition given in Sect. 2.3.

If $D = D_1 \cup \cdots \cup D_n$ is the union of a finite number of disjoint subsets of $\hat{\mathbb {C}}$ each of which is bounded by a curve in $\mathcal {J}$, we define $E^p(D)$ and $\dot{E}^p(D)$ as the set of functions *f* analytic in *D* such that $f|_{D_j} \in E^p(D_j)$ and $f|_{D_j} \in \dot{E}^p(D_j)$ for each *j*, respectively.

### Properties of $E^p(D)$ and $\dot{E}^p(D)$

Our definitions of the generalized Smirnoff classes $E^p(D)$ and $\dot{E}^p(D)$ are designed in such a way that these classes possess convenient transformation properties under the action of the group of linear fractional transformations.

#### Proposition 3.6

Let $1 \le p < \infty$. Let $\Gamma$ be a Carleson jump contour and let *D* be the union of any number of components of $\hat{\mathbb {C}}{\setminus }\Gamma$. Let $\psi (z)$ be an arbitrary linear fractional transformation of the form (3.1) with $ad - bc \ne 0$.

- $f \in E^p(D)$ if and only if $f \circ \psi ^{-1} \in E^p(\psi (D))$.
- $f \in \dot{E}^p(D)$ if and only if $\Psi f \in \dot{E}^p(\psi (D))$ where $(\Psi f)(w) = (cw - a)^{-1} f(\psi ^{-1}(w))$.

#### Proof

Without loss of generality, we may assume that *D* is one of the components of $\hat{\mathbb {C}}{\setminus }\Gamma$ where $\Gamma \in \mathcal {J}$.

(*a*): We will prove that $f \in E^p(D)$ if and only if $f \circ \psi ^{-1} \in E^p(\psi (D))$ whenever $\psi (D)$ is bounded and either (*i*) $\infty \in D$, (*ii*) *D* is bounded, or (*iii*) $\infty \in \Gamma$. Since the linear fractional transformations form a group, this will prove (*a*). *Case (i).* Suppose $\psi (D)$ is bounded, $\infty \in D$, and $f \in E^p(D)$. By the definition of $E^p(D)$ in Sect. 2.3, there exists a sequence of rectifiable Jordan curves $\{C_n\}_1^\infty$ in *D* tending to the boundary $\Gamma$ such that (2.5) holds. It follows that $\{\psi (C_n)\}_1^\infty$ is a sequence of rectifiable Jordan curves in $\psi (D)$ tending to $\psi (\Gamma )$ and
  3.4 $$\begin{aligned} \sup _{n \ge 1} \int _{\psi (C_n)} |f(\psi ^{-1}(w))|^p |dw| = \sup _{n \ge 1} \int _{C_n} |f(z)|^p |\psi '(z)| |dz|. \end{aligned}$$
  If $c = 0$, then $\psi '(z)$ is a finite constant. If $c \ne 0$, then our assumption that $\psi (D)$ is bounded implies that the point $z = -d/c$ does not belong to $\bar{D}$; hence the function
  $$\begin{aligned} \psi '(z) = \frac{ad - bc}{(cz + d)^2} \end{aligned}$$
  is bounded on *D*. It follows that the right-hand side of (3.4) is finite. Thus $f \circ \psi ^{-1} \in E^p(\psi (D))$. Conversely, if $\psi (D)$ is bounded, $\infty \in D$, and $f \circ \psi ^{-1} \in E^p(\psi (D))$, then a similar argument shows that $f \in E^p(D)$. *Case (ii).* Suppose both $\psi (D)$ and *D* are bounded. An argument similar to that used in Case (*i*) shows that $f \in E^p(D)$ if and only if $f \circ \psi ^{-1} \in E^p(\psi (D))$. *Case (iii).* Suppose $\psi (D)$ is bounded and $\infty \in \Gamma$. Let $z_0 \in \mathbb {C}{\setminus }(D \cup \Gamma )$. By the definition of $E^p(D)$, $f \in E^p(D)$ if and only if $f \circ \varphi ^{-1} \in E^p(\varphi (D))$, where $\varphi (z) = 1/(z - z_0)$. But $\psi \circ \varphi ^{-1}$ is a linear fractional transformation mapping the bounded domain $\varphi (D)$ onto the bounded domain $\psi (D)$; hence Case (*ii*) implies that $f \circ \varphi ^{-1} \in E^p(\varphi (D))$ if and only if $f \circ \varphi ^{-1} \circ (\psi \circ \varphi ^{-1})^{-1} = f \circ \psi ^{-1}$ belongs to $E^p(\psi (D))$. This completes the proof of (*a*).
(*b*): This part is a consequence of (*a*) and the definitions. Indeed, suppose first that $c \ne 0$. By definition of $\dot{E}^p(D)$, $f \in \dot{E}^p(D)$ if and only if $f(z), zf(z) \in E^p(D)$. Since $E^p(D)$ is a linear space, this is the case if and only if $f(z), (cz + d)f(z) \in E^p(D)$. Using (*a*) and the fact that $cz + d = \frac{bc - ad}{cw - a}$ when $w = \psi (z)$, the latter condition is equivalent to $f(\psi ^{-1}(w)), \frac{bc - ad}{cw - a}f(\psi ^{-1}(w)) \in E^p(\psi (D))$. Using that $E^p(D)$ is a linear space again, this holds if and only if $(cw - a)^{-1} f(\psi ^{-1}(w)) \in \dot{E}^p(\psi (D))$. The proof when $c = 0$ is similar. This proves (*b*).

$$\square $$

It is possible to characterize the spaces $E^p(D)$ and $\dot{E}^p(D)$ in terms of conditions analogous to (2.5) also when $\infty \in \partial D$.

#### Lemma 3.7

Let $1 \le p < \infty$. Let *D* be a subset of $\hat{\mathbb {C}}$ bounded by a curve $\Gamma \in \mathcal {J}$ with $\infty \in \Gamma$. Let $z_0 \in \mathbb {C}{\setminus }\bar{D}$ and let $f:D \rightarrow \mathbb {C}$ be an analytic function. Then

- $f \in E^p(D)$ if and only if there exist curves $\{C_n\}_1^\infty \subset \mathcal {J}$ in *D*, tending to $\Gamma$ in the sense that $C_n$ eventually surrounds each compact subset of *D*, such that
  3.5 $$\begin{aligned} \sup _{n \ge 1} \int _{C_n} |z - z_0|^{-2} |f(z)|^p |dz| < \infty . \end{aligned}$$
- $f \in \dot{E}^p(D)$ if and only if there exist curves $\{C_n\}_1^\infty \subset \mathcal {J}$ in *D*, tending to $\Gamma$ in the sense that $C_n$ eventually surrounds each compact subset of *D*, such that
  3.6 $$\begin{aligned} \sup _{n \ge 1} \int _{C_n} |z - z_0|^{p-2} |f(z)|^p |dz| < \infty . \end{aligned}$$

#### Proof

Let $\varphi (z) = \frac{1}{z-z_0}$. If $\{C_n\}_1^\infty \subset \mathcal {J}$ are curves in *D* tending to $\Gamma$, then $\{\varphi (C_n)\}_1^\infty$ are curves in $\varphi (D)$ tending to $\varphi (\Gamma )$. Each $\varphi (C_n)$ is a rectifiable Jordan curve, because $\varphi (C_n) \in \mathcal {J}$ by Proposition 3.1. The change of variables $w = \varphi (z)$ gives

3.7 $$\begin{aligned} \sup _{n \ge 1} \int _{\varphi (C_n)} |f(\varphi ^{-1}(w))|^p |dw| = \sup _{n \ge 1} \int _{C_n} |z - z_0|^{-2} |f(z)|^p |dz| \end{aligned}$$

and

3.8 $$\begin{aligned} \sup _{n \ge 1} \int _{\varphi (C_n)} |w^{-1} f(\varphi ^{-1}(w))|^p |dw| = \sup _{n \ge 1} \int _{C_n} |z - z_0|^{p-2} |f(z)|^p |dz|. \end{aligned}$$

If (3.5) holds, then (3.7) gives $f \circ \varphi ^{-1} \in E^p(\varphi (D))$; hence $f \in E^p(D)$ by Proposition 3.6 (*a*). If (3.6) holds, then (3.8) gives $w^{-1} f(\varphi ^{-1}(w)) \in E^p(\varphi (D))$; hence $f \in \dot{E}^p(D)$ by Proposition 3.6 (*b*).

It remains to prove the opposite implications. Let $u \mapsto \psi (u)$ be a conformal isomorphism from the open unit disk onto $\varphi (D)$ and let $\Gamma _r$ be the image under $\psi$ of the circle $|u| = r$. Suppose $f \in E^p(D)$. Then Proposition 3.6 (*a*) implies that $f \circ \varphi ^{-1} \in E^p(\varphi (D))$. Hence, by Theorem 10.1 in [14],

$$\begin{aligned} \sup _{r< 1}\int _{\Gamma _r} |f(\varphi ^{-1}(w))|^p |dw| < \infty . \end{aligned}$$

Applying the change of variables $w = \varphi (z)$, we find

$$\begin{aligned} \sup _{r< 1}\int _{C_r} |z - z_0|^{-2} |f(z)|^p |dz| < \infty . \end{aligned}$$

where $C_r = \varphi ^{-1}(\Gamma _r)$. For each $r < 1$, the curve $C_r$ belongs to $\mathcal {J}$, because it is the image of the circle $|u| = r$ under the conformal bijection $\varphi ^{-1} \circ \psi$ (see Remark 3.2). Thus the proof of (*a*) is complete. A similar argument applies if $f \in \dot{E}^p(D)$. Indeed, suppose $f \in \dot{E}^p(D)$. Then Proposition 3.6 (*b*) implies that $w^{-1} f(\varphi ^{-1}(w)) \in E^p(\varphi (D))$. Hence, by the change of variables $w = \varphi (z)$ and Theorem 10.1 in [14],

$$\begin{aligned} \sup _{r< 1}\int _{C_r} |z - z_0|^{p-2} |f(z)|^p |dz| = \sup _{r< 1}\int _{\Gamma _r} |w^{-1} f(\varphi ^{-1}(w))|^p |dw| < \infty . \end{aligned}$$

This completes the proof of (*b*). $\square$

#### Lemma 3.8

Let *D* be a subset of $\hat{\mathbb {C}}$ bounded by a curve in $\mathcal {J}$.

- $$\dot{E}^p(D) \subset \dot{E}^r(D)$$ whenever $$1 \le r \le p < \infty $$.
- Suppose $p,q,r \in [1, \infty )$ satisfy $1/p + 1/q = 1/r$. If $f \in \dot{E}^p(D)$ and $g \in \dot{E}^q(D)$, then the functions *zf*(*z*)*g*(*z*) and *fg* belong to $\dot{E}^r(D)$.

#### Proof

(*a*): The result is immediate from the definitions if $\infty \notin \Gamma$. Thus suppose $\infty \in \Gamma$. Let $z_0 \in \mathbb {C}{\setminus }\Gamma$ and define $\varphi$ as in (3.3). If $f \in \dot{E}^p(D)$, then Proposition 3.6 implies that $w^{-1}f(\varphi ^{-1}(w)) \in \dot{E}^p(\varphi (D))$; since $\dot{E}^p(\varphi (D)) \subset \dot{E}^r(\varphi (D))$, another application of Proposition 3.6 shows that $f \in \dot{E}^r(D)$. This proves (*a*).
(*b*): Suppose $p,q,r \in [1, \infty )$ satisfy $1/p + 1/q = 1/r$. Let $f \in \dot{E}^p(D)$ and $g \in \dot{E}^q(D)$. We first suppose *D* is bounded. Then there exist sequences of rectifiable Jordan curves $\{A_n\}_1^\infty$ and $\{B_n\}_1^\infty$ in *D* tending to the boundary of *D* such that
  $$\begin{aligned} \sup _{n \ge 1} \Vert f\Vert _{L^p(A_n)}< \infty , \qquad \sup _{n \ge 1} \Vert g\Vert _{L^q(B_n)} < \infty . \end{aligned}$$
  Without loss of generality, we may assume that $A_n = B_n = C_n$ for all $n \ge 1$ where $\{C_n\}_1^\infty$ are level curves of an arbitrary conformal map of the unit disk onto *D* (see Theorem 10.1 in [14]). Then, by Hölder’s inequality,
  $$\begin{aligned} \sup _{n \ge 1} \Vert fg\Vert _{L^r(C_n)} \le \sup _{n \ge 1} \Vert f\Vert _{L^p(C_n)} \Vert g\Vert _{L^q(C_n)} < \infty . \end{aligned}$$
  Hence $fg \in E^r(D) = \dot{E}^r(D)$. This proves (*b*) when *D* is bounded. If *D* is unbounded, then pick $z_0 \in \mathbb {C}{\setminus }(D \cup \Gamma )$ and let $\varphi (z) = 1/(z - z_0)$. By Proposition 3.6, $w^{-1} f(\varphi ^{-1}(w)) \in \dot{E}^p(\varphi (D))$ and $w^{-1} g(\varphi ^{-1}(w)) \in \dot{E}^q(\varphi (D))$. Hence, by the preceding paragraph, $w^{-2}(fg)(\varphi ^{-1}(w)) = w^{-1}(fg/\varphi )(\varphi ^{-1}(w)) \in \dot{E}^r(\varphi (D))$. Since $\varphi (D)$ is bounded, we also have $w^{-1}(fg)(\varphi ^{-1}(w)) \in \dot{E}^r(\varphi (D))$. Using Proposition 3.6 again, we conclude that $fg/\varphi , fg \in \dot{E}^r(D)$. Part (*b*) follows.

$$\square $$

### The spaces $\dot{L}^p(\Gamma )$

Let $1 \le p < \infty$. Let $\Gamma$ be a Carleson curve. We define $\dot{L}^p(\Gamma )$ as the set of all measurable functions on $\Gamma$ such that $|z - z_0|^{1 - \frac{2}{p}}h(z) \in L^p(\Gamma )$ for some (and hence all) $z_0 \in \mathbb {C}{\setminus } \Gamma$. Note that

$$\begin{aligned}&\dot{L}^p(\Gamma ) \subset L^p(\Gamma ), \qquad 2 \le p < \infty ,\\&L^p(\Gamma ) \subset \dot{L}^p(\Gamma ), \qquad 1 \le p \le 2, \end{aligned}$$

If $h \in \dot{L}^p(\Gamma )$, then the value of the Cauchy integral $(\mathcal {C}h)(z)$ is well-defined for all $z \in \mathbb {C}{\setminus } \Gamma$. Indeed, if $1/p + 1/q = 1$, then the Carleson property of $\Gamma$ implies $\Vert |\cdot - z|^{- \frac{2}{q}} \Vert _{L^q(\Gamma )} < \infty$; hence, by Hölder’s inequality,

$$\begin{aligned} \int _\Gamma \frac{|h(z')|}{|z' - z|}|dz'| \le \big \Vert |\cdot - z|^{1 - \frac{2}{p}} h \big \Vert _{L^p(\Gamma )} \big \Vert |\cdot - z|^{- \frac{2}{q}} \big \Vert _{L^q(\Gamma )} < \infty . \end{aligned}$$

If $\Gamma$ is bounded, then $\dot{L}^p(\Gamma ) = L^p(\Gamma )$.

#### Lemma 3.9

Let $1 \le p < \infty$ and let $\Gamma$ be a Carleson curve. Let $z_0 \in \mathbb {C}{\setminus } \Gamma$ and let $\varphi$ be given by (3.3).

- The map $\Phi$ defined for $h \in \dot{L}^p(\Gamma )$ by
  3.9 $$\begin{aligned} (\Phi h)(w) = w^{-1} h(\varphi ^{-1}(w)) \end{aligned}$$
  is a bijection $\dot{L}^p(\Gamma ) \rightarrow L^p(\varphi (\Gamma ))$ and
  $$\begin{aligned} \Vert |\cdot - z_0|^{1-\frac{2}{p}} h\Vert _{L^p(\Gamma )} = \Vert \Phi h\Vert _{L^p(\varphi (\Gamma ))} \end{aligned}$$
  for all $$h \in \dot{L}^p(\Gamma )$$.
- If $$h \in \dot{L}^p(\Gamma )$$, then
  3.10 $$\begin{aligned} (\mathcal {C}_\Gamma h)(z) = (\Psi ^{-1} \mathcal {C}_{\varphi (\Gamma )} \Phi h)(z) \quad \text {for all }z \in \mathbb {C}{\setminus } \Gamma , \end{aligned}$$
  where $\Psi$ acts on a function $f:\hat{\mathbb {C}}{\setminus } \Gamma \rightarrow \mathbb {C}$ by $(\Psi f)(w) = w^{-1} f(\varphi ^{-1}(w))$.

#### Proof

(*a*): If $h \in \dot{L}^p(\Gamma )$, then the change of variables $w = \varphi (z)$ implies
  $$\begin{aligned} \Vert |z-z_0|^{1-\frac{2}{p}} h(z)\Vert _{L^p(\Gamma )}^p&= \int _\Gamma |z-z_0|^{p-2} |h(z)|^p |dz|\\&= \int _{\varphi (\Gamma )} |w^{-1} h(\varphi ^{-1}(w))|^p |dw| = \Vert \Phi h\Vert _{L^p(\varphi (\Gamma ))}^p. \end{aligned}$$
  We infer that $\Phi$ is a bijection $\dot{L}^p(\Gamma ) \rightarrow L^p(\varphi (\Gamma ))$ with inverse given by $(\Phi ^{-1}H)(z) = \varphi (z) H(\varphi (z))$. This proves (*a*).
(*b*): If $h \in \dot{L}^p(\Gamma )$, $z \in \mathbb {C}{\setminus } \Gamma$ and $w = \varphi (z)$, then
  3.11 $$\begin{aligned} (\mathcal {C}_{\varphi (\Gamma )} \Phi h)(w)&= \frac{1}{2\pi i} \int _{\varphi (\Gamma )} \frac{(h \circ \varphi ^{-1})(w')}{w' - w}\frac{dw'}{w'} \nonumber \\&= \frac{z - z_0}{2\pi i} \int _{\Gamma } \frac{h(z')}{z' - z} dz' = (z-z_0) (\mathcal {C}_\Gamma h)(z) = \Psi (\mathcal {C}_\Gamma h)(w), \end{aligned}$$
  which proves (*b*).

$$\square $$

For each $z_0 \in \mathbb {C}{\setminus } \Gamma$, we define a norm on $\dot{L}^p(\Gamma )$ by

3.12 $$\begin{aligned} \Vert h\Vert _{\dot{L}^p(\Gamma ), z_0} = \Vert |\cdot - z_0|^{1 - \frac{2}{p}} h\Vert _{L^p(\Gamma )}. \end{aligned}$$

The space $\dot{L}^p(\Gamma )$ with the norm $\Vert \cdot \Vert _{\dot{L}^p(\Gamma ), z_0}$ is nothing but the weighted space $L^p(\Gamma , w)$ with $w(z) = |z - z_0|^{1 - \frac{2}{p}}$. Different choices of $z_0 \in \mathbb {C}{\setminus } \Gamma$ induce different norms on $\dot{L}^p(\Gamma )$, but these norms are all equivalent. We say that an operator *T* on $\dot{L}^p(\Gamma )$ is bounded if it is bounded with respect to one (and hence all) of these norms.

#### Lemma 3.10

Let $$\Gamma \in \mathcal {J}$$.

- $$\dot{L}^p(\Gamma ) \subset \dot{L}^r(\Gamma )$$ whenever $$1 \le r \le p < \infty $$.
- Suppose $p,q,r \in [1, \infty )$ satisfy $1/p + 1/q = 1/r$. If $f \in \dot{L}^p(\Gamma )$ and $g \in \dot{L}^q(\Gamma )$, then the functions *zf*(*z*)*g*(*z*) and *fg* belong to $\dot{L}^r(\Gamma )$.

#### Proof

(*a*): The result is immediate from the definitions if $\infty \notin \Gamma$. Thus suppose $\infty \in \Gamma$. Let $z_0 \in \mathbb {C}{\setminus } \Gamma$ and define $\varphi$ as in (3.3). If $h \in \dot{L}^p(\Gamma )$, then Lemma 3.9 implies that $w^{-1}h(\varphi ^{-1}(w)) \in \dot{L}^p(\varphi (\Gamma ))$; since $\dot{L}^p(\varphi (\Gamma )) \subset \dot{L}^r(\varphi (\Gamma ))$, another application of Lemma 3.9 shows that $h \in \dot{L}^r(\Gamma )$. This proves (*a*).
(*b*): Suppose $p,q,r \in [1, \infty )$ satisfy $1/p + 1/q = 1/r$. Let $f \in \dot{L}^p(\Gamma )$ and $g \in \dot{L}^q(\Gamma )$. Suppose $\infty \notin \Gamma$. Then, by Hölder’s inequality,
  $$\begin{aligned} \Vert fg\Vert _{L^r(\Gamma )} \le \Vert f\Vert _{L^p(\Gamma )} \Vert g\Vert _{L^q(\Gamma )} < \infty . \end{aligned}$$
  Hence $fg \in L^r(\Gamma ) = \dot{L}^r(\Gamma )$. This gives (*b*) when $\infty \notin \Gamma$. If $\infty \in \Gamma$, then pick $z_0 \in \mathbb {C}{\setminus } \Gamma$ and define $\varphi$ as in (3.3). By Lemma 3.9, $w^{-1} f(\varphi ^{-1}(w)) \in L^p(\varphi (\Gamma ))$ and $w^{-1} g(\varphi ^{-1}(w)) \in L^q(\varphi (\Gamma ))$. Hence, by the preceding paragraph, $w^{-2}(fg)(\varphi ^{-1}(w)) = w^{-1}(fg/\varphi )(\varphi ^{-1}(w)) \in L^r(\varphi (\Gamma ))$. Since $\varphi (\Gamma )$ is bounded, we also have $w^{-1}(fg)(\varphi ^{-1}(w)) \in L^r(\varphi (\Gamma ))$. Using Lemma 3.9 again, we conclude that $fg/\varphi , fg \in \dot{L}^r(\Gamma )$. Part (*b*) follows.

$$\square $$

### The Cauchy singular operator

Theorem 2.1 can be used to establish boundedness of the Cauchy singular operator $\mathcal {S}_\Gamma$ on $\dot{L}^p(\Gamma )$ if $1< p < \infty$ and $\Gamma$ is Carleson.

#### Proposition 3.11

Let $1< p < \infty$ and let $\Gamma$ be a Carleson curve. Then $\mathcal {S}_\Gamma$ generates a bounded operator $\tilde{\mathcal {S}}_\Gamma$ on $\dot{L}^p(\Gamma )$. Moreover, if $h \in \dot{L}^p(\Gamma )$, then the limit in (2.3) exists and $(\mathcal {S}_\Gamma h)(z) = (\tilde{\mathcal {S}}_\Gamma h)(z)$ for a.e. $z \in \Gamma$.

#### Proof

Let $z_0 \notin \Gamma$ and let $w(z) = |z - z_0|^{1 - \frac{2}{p}}$. The result follows from Theorem 2.1 if we can show that $w \in A_p(\Gamma )$. If $p =2$, this is an immediate consequence of $\Gamma$ being Carleson. Thus suppose $p \ne 2$.

Define *q* by $1/p + 1/q = 1$ and let $z \in \Gamma$. If $0 < r \le \frac{|z-z_0|}{2}$ and $z' \in D(z,r)$, then

$$\begin{aligned} \frac{|z - z_0|}{2} \le |z' - z_0| \le \frac{3|z - z_0|}{2} . \end{aligned}$$

Using the Carleson condition (2.1) on the disk *D*(*z*, *r*), we find that there exists a constant $C_\alpha > 0$ depending only on $\alpha$ such that

3.13a $$\begin{aligned}&\frac{1}{r} \int _{\Gamma \cap D(z, r)} |z' - z_0|^\alpha |dz'| \le \frac{1}{r} \frac{3^\alpha |z-z_0|^\alpha }{2^\alpha } |\Gamma \cap D(z, r)| \le C_\alpha |z-z_0|^\alpha , \end{aligned}$$

3.13b $$\begin{aligned}&\frac{1}{r} \int _{\Gamma \cap D(z, r)} |z' - z_0|^{-\alpha } |dz'| \le \frac{1}{r} \frac{|z-z_0|^{-\alpha }}{2^{-\alpha }} |\Gamma \cap D(z, r)| \le C_\alpha |z-z_0|^{-\alpha }, \end{aligned}$$

whenever $0 < r \le \frac{|z-z_0|}{2}$ and $\alpha > 0$. This yields

3.14 $$\begin{aligned}&\sup _{z \in \Gamma } \sup _{0< r \le \frac{|z-z_0|}{2}} \bigg (\frac{1}{r} \int _{\Gamma \cap D(z, r)} w(z')^p |dz'|\bigg )^{1/p}\bigg (\frac{1}{r} \int _{\Gamma \cap D(z, r)} w(z')^{-q} |dz'|\bigg )^{1/q} \nonumber \\&\quad = \sup _{z \in \Gamma } \sup _{0< r \le \frac{|z-z_0|}{2}} \bigg (\frac{1}{r} \int _{\Gamma \cap D(z, r)} |z' - z_0|^{p -2} |dz'|\bigg )^{1/p}\nonumber \\&\qquad \times \bigg (\frac{1}{r} \int _{\Gamma \cap D(z, r)} |z' - z_0|^{\frac{q}{p} - 1} |dz'|\bigg )^{1/q} \nonumber \\&\quad \le C_p \sup _{z \in \Gamma } \bigg (|z-z_0|^{p - 2} \bigg )^{1/p}\bigg (|z-z_0|^{\frac{q}{p} - 1} \bigg )^{1/q} = C_p < \infty , \end{aligned}$$

with $C_p > 0$ depending only on *p*.

On the other hand, if $R = |z - z_0| + r$, then the Carleson condition on the disk $D(z_0, 2^{1-n}R)$ yields

3.15 $$\begin{aligned} \frac{1}{r} \int _{\Gamma \cap D(z, r)} |z' - z_0|^\alpha |dz'|&=\frac{1}{r} \sum _{n=1}^\infty \int _{\Gamma \cap D(z, r) \cap (D(z_0, 2^{1-n}R) {\setminus } D(z_0, 2^{-n}R))} |z' - z_0|^\alpha |dz'| \nonumber \\&\le \frac{1}{r} \sum _{n=1}^\infty 2^{(1-n)\alpha }R^\alpha |\Gamma \cap (D(z_0, 2^{1-n}R) {\setminus } D(z_0, 2^{-n}R))| \nonumber \\&\le \frac{C R^{1+ \alpha } }{r} \sum _{n=1}^\infty 2^{(1-n)\alpha } 2^{1-n} \le \frac{2C (|z - z_0| + r)^{1 + \alpha }}{r} \nonumber \\&\le C_\alpha r^\alpha \end{aligned}$$

whenever $r > \frac{|z-z_0|}{2}$ and $\alpha > 0$. Similarly,

3.16 $$\begin{aligned}&\frac{1}{r} \int _{\Gamma \cap D(z, r)} |z' - z_0|^{-\alpha } |dz'| \nonumber \\&\quad =\frac{1}{r} \sum _{n=1}^\infty \int _{\Gamma \cap D(z, r) \cap (D(z_0, 2^{1-n}R) {\setminus } D(z_0, 2^{-n}R))} |z' - z_0|^{-\alpha } |dz'| \nonumber \\&\quad \le \frac{1}{r} \sum _{n=1}^\infty 2^{n\alpha }R^{-\alpha } |\Gamma \cap (D(z_0, 2^{1-n}R) {\setminus } D(z_0, 2^{-n}R))| \nonumber \\&\quad \le \frac{C R^{1- \alpha } }{r} \sum _{n=1}^\infty 2^{n\alpha } 2^{1-n} \le \frac{C_\alpha (|z - z_0| + r)^{1 - \alpha }}{r} \le C_\alpha r^{- \alpha }, \end{aligned}$$

whenever $r > \frac{|z-z_0|}{2}$ and $0< \alpha < 1$. If $p > 2$, we apply (3.15) with $\alpha = p - 2$ and (3.16) with $\alpha = 1- \frac{q}{p}$. If $1< p < 2$, we apply (3.16) with $\alpha = 2- p$ and (3.15) with $\alpha = \frac{q}{p} - 1$. This yields

3.17 $$\begin{aligned}&\sup _{z \in \Gamma } \sup _{r> \frac{|z-z_0|}{2}} \bigg (\frac{1}{r} \int _{\Gamma \cap D(z, r)} w(z')^p |dz'|\bigg )^{1/p}\bigg (\frac{1}{r} \int _{\Gamma \cap D(z, r)} w(z')^{-q} |dz'|\bigg )^{1/q} \nonumber \\&\quad \le C \sup _{z \in \Gamma } \sup _{r > \frac{|z-z_0|}{2}} \big (r^{p -2} \big )^{1/p} \big (r^{\frac{q}{p} - 1} \big )^{1/q} = C < \infty . \end{aligned}$$

It follows from (3.14) and (3.17) that *w*(*z*) satisfies the Muckenhoupt condition (2.4). $\square$

Our next objective is to determine how $\mathcal {S}_\Gamma$ transforms under the change of variables $w = 1/(z - z_0)$. We need the following lemma.

#### Lemma 3.12

Let $\Gamma$ be a Carleson jump contour. Let $g(\epsilon )$ be a nondecreasing continuous function of $\epsilon \ge 0$ such that $g(0) = 0$. If $z \in \Gamma \cap \mathbb {C}$ is a point at which $\Gamma$ has a two-sided tangent, then the following limit exists and equals zero:

3.18 $$\begin{aligned} \lim _{\epsilon \rightarrow 0} \int _{\Gamma \cap [D(z, \epsilon (1 + g(\epsilon ))) {\setminus } D(z, \epsilon (1 - g(\epsilon )))]}\frac{|dz'|}{|z' - z|} = 0. \end{aligned}$$

#### Proof

Without loss of generality, we assume that $\Gamma \in \mathcal {J}$. Let $z \in \Gamma \cap \mathbb {C}$ be a point at which $\Gamma$ has a two-sided tangent. Let $\gamma (s)$, $-s_0< s < s_0$, be an arclength parametrization of $\Gamma$ in a neighborhood of $\gamma (0) = z$. Suppose without loss of generality that $\gamma '(0) = 1$. Then

$$\begin{aligned} \gamma (s) = z + s + o(|s|), \qquad s \rightarrow 0. \end{aligned}$$

For each $r \in (0,1/2]$, choose $\delta (r) \in (0, s_0)$ such that

3.19 $$\begin{aligned} |o(|s|)| < r |s| \quad \text {for} \quad |s| \le \delta (r). \end{aligned}$$

We may assume that $\delta (r)$ is a nondecreasing function of $r > 0$. Replacing $\delta (r)$ with $\int _0^r \delta (t)dt \le \delta (r)$ if necessary (note that all nondecreasing functions are measurable), we may assume that $\delta (r)$ is a continuous strictly increasing function such that $\lim _{r \rightarrow 0^+} \delta (r) = 0$. Let $\gamma _{1/2}$ denote the subarc

$$\begin{aligned} \gamma _{1/2} = \{\gamma (s) | |s| \le \delta (1/2)\} \end{aligned}$$

and let *a*, *b* be the endpoints of $\gamma _{1/2}$. The set $(\Gamma {\setminus } \gamma _{1/2}) \cup \{a, b\}$ is compact. Let $\mu$ be the minimum of the continuous function $|\cdot - z|$ on this set. Then $\Gamma \cap D(z, \mu ) \subset \gamma _{1/2}$. Fix $r\in (0,1/2]$ such that $\delta (r) < \mu$. We claim that

3.20 $$\begin{aligned} |s| < \frac{\delta (r)}{2(1-r)}, \end{aligned}$$

whenever $\gamma (s) \in \gamma _{1/2}$ and $|\gamma (s) - z| \le \frac{\delta (r)}{2}$. Indeed, suppose $|s| \le \delta (1/2)$ is such that $|\gamma (s) - z| \le \frac{\delta (r)}{2}$. Then (3.19) implies

$$\begin{aligned} \frac{|s|}{2} < |s + o(|s|)| = |\gamma (s) - z| \le \frac{\delta (r)}{2}. \end{aligned}$$

Thus $|s| < \delta (r)$, so another application of (3.19) yields

$$\begin{aligned} (1 - r) |s| < |s + o(|s|)| = |\gamma (s) - z| \le \frac{\delta (r)}{2}, \end{aligned}$$

which proves (3.20). On the other hand, since *s* is an arclength parameter,

3.21 $$\begin{aligned} |s| \ge \frac{\delta (r)}{2}\bigg (1 - g\bigg (\frac{\delta (r)}{2}\bigg )\bigg ), \end{aligned}$$

whenever $$|\gamma (s) - z| \ge \frac{\delta (r)}{2}(1 - g(\frac{\delta (r)}{2}))$$.

For $\epsilon > 0$, we define the closed annulus $K(z, \epsilon )$ by

$$\begin{aligned} K(z, \epsilon ) = \overline{D(z, \epsilon )} {\setminus } D(z, \epsilon (1 - g(\epsilon ))). \end{aligned}$$

Then the set $\{s \in [0,\delta (1/2)] | \gamma (s) \in K(z, \delta (r)/2)\}$ is closed. Let $s_+ \ge 0$ and $s_- \ge 0$ denote the largest and smallest elements of this set, respectively. Clearly,

$$\begin{aligned} |\gamma (s_+) - z| \le \frac{\delta (r)}{2}, \qquad |\gamma (s_-) - z| \ge \frac{\delta (r)}{2}\bigg (1 - g\bigg (\frac{\delta (r)}{2}\bigg )\bigg ). \end{aligned}$$

Hence, by (3.20) and (3.21),

$$\begin{aligned} \frac{\delta (r)}{2}\bigg (1 - g\bigg (\frac{\delta (r)}{2}\bigg )\bigg ) \le s_- \le s_+ < \frac{\delta (r)}{2(1-r)}. \end{aligned}$$

Thus

$$\begin{aligned} \bigg |\gamma ([0,\delta (1/2)]) \cap K\bigg (z, \frac{\delta (r)}{2}\bigg )\bigg | \le s_+ - s_- < \frac{\delta (r)}{2} \bigg ( \frac{r}{1-r} + g\bigg (\frac{\delta (r)}{2}\bigg )\bigg ). \end{aligned}$$

A similar argument shows that

$$\begin{aligned} \bigg |\gamma ([-\delta (1/2), 0]) \cap K\bigg (z, \frac{\delta (r)}{2}\bigg )\bigg | < \frac{\delta (r)}{2} \bigg ( \frac{r}{1-r} + g\bigg (\frac{\delta (r)}{2}\bigg )\bigg ). \end{aligned}$$

Consequently, for all small enough $r > 0$,

3.22 $$\begin{aligned} \bigg |\Gamma \cap K\bigg (z, \frac{\delta (r)}{2}\bigg )\bigg | = \bigg |\gamma _{1/2} \cap K\bigg (z, \frac{\delta (r)}{2}\bigg )\bigg | < F(r) \end{aligned}$$

where the function

$$\begin{aligned} F(r) = \delta (r) \bigg ( \frac{r}{1-r} + g\bigg (\frac{\delta (r)}{2}\bigg )\bigg ) \end{aligned}$$

satisfies

$$\begin{aligned} \lim _{r \rightarrow 0^+} \frac{F(r)}{\delta (r)} = 0. \end{aligned}$$

Given $\epsilon >0$ small enough, there exists a unique $r = r(\epsilon ) > 0$ such that $\delta (r)/2 = \epsilon$. It follows that

$$\begin{aligned} \int _{\Gamma \cap K(z,\epsilon )}\frac{|dz'|}{|z' - z|} \le \frac{|\Gamma \cap K(z,\epsilon )|}{\epsilon (1 - g(\epsilon ))} < \frac{2 F(r)}{\delta (r) (1 - g(\epsilon ))} \rightarrow 0 \end{aligned}$$

as $\epsilon \rightarrow 0$. This proves that

3.23 $$\begin{aligned} \lim _{\epsilon \rightarrow 0} \int _{\Gamma \cap [D(z, \epsilon ) {\setminus } D(z, \epsilon (1 - g(\epsilon )))]}\frac{|dz'|}{|z' - z|} = 0. \end{aligned}$$

Equation (3.18) follows from (3.23) by changing variables $\tilde{\epsilon } = \epsilon (1 + g(\epsilon ))$ in the left-hand side of (3.18) and noting that $\epsilon (1 - g(\epsilon )) = \tilde{\epsilon }(1 - \tilde{g}(\tilde{\epsilon }))$, where $\tilde{g}(\tilde{\epsilon }) = \frac{2g(\epsilon )}{1 + g(\epsilon )}$ is a continuous nondecreasing function of $\tilde{\epsilon }$. $\square$

#### Proposition 3.13

Let $1< p < \infty$ and let $\Gamma$ be a Carleson jump contour. Let $z_0 \in \mathbb {C}{\setminus } \Gamma$ and let $\varphi (z) = 1/(z-z_0)$. Let $\Phi :\dot{L}^p(\Gamma ) \rightarrow L^p(\varphi (\Gamma ))$ be the bijection defined in (3.9). Then

3.24 $$\begin{aligned} \mathcal {S}_\Gamma h = \Phi ^{-1} \mathcal {S}_{\varphi (\Gamma )} \Phi h \quad \text {a.e. on }\Gamma \end{aligned}$$

for every $h \in \dot{L}^p(\Gamma )$. In other words, the following diagram commutes:


#### Proof

We will show that $\mathcal {S}_\Gamma h = \Phi ^{-1} \mathcal {S}_{\varphi (\Gamma )} \Phi h$ a.e. on $\Gamma$ whenever $h \in C_0^\infty (\Gamma )$. Since $C_0^\infty (\Gamma )$ is dense in $\dot{L}^p(\Gamma )$ and the operators $\mathcal {S}_\Gamma$ and $\Phi ^{-1} \mathcal {S}_{\varphi (\Gamma )} \Phi$ are bounded on $\dot{L}^p(\Gamma )$ by Lemma 3.9 and Proposition 3.11, this will prove (3.24).

Let $h \in C_0^\infty (\Gamma )$. A change of variables shows that

3.25 $$\begin{aligned} \frac{1}{\pi i} \int _{\Gamma {\setminus } D(z, \epsilon )} \frac{h(z')}{z' - z} dz' = \frac{\varphi (z)}{\pi i} \int _{\varphi (\Gamma ) {\setminus } \varphi (D(z, \epsilon ))} \frac{(\Phi h)(w')}{w' - \varphi (z)} dw' \end{aligned}$$

for all $z \in \Gamma \cap \mathbb {C}$ and $\epsilon > 0$. As $\epsilon \rightarrow 0$, the left-hand side of (3.25) tends to $(\mathcal {S}_\Gamma h)(z)$ for a.e. $z \in \Gamma$. It remains to prove that the right-hand side of (3.25) tends to

3.26 $$\begin{aligned} (\Phi ^{-1} \mathcal {S}_{\varphi (\Gamma )} \Phi h)(z) = \varphi (z) (\mathcal {S}_{\varphi (\Gamma )} \Phi h)(\varphi (z)) \end{aligned}$$

for a.e. $z \in \Gamma$ as $\epsilon \rightarrow 0$. The proof of this fact is complicated by the fact that, in general, the disk $\varphi (D(z, \epsilon ))$ is not centered at $\varphi (z)$.

Let $z \in \Gamma$ and let $0< \epsilon < |z-z_0|$. Then

$$\begin{aligned} \varphi (D(z,\epsilon )) = D\bigg ( \frac{\bar{z} - \bar{z}_0}{|z - z_0|^2 - \epsilon ^2}, \tilde{\epsilon }\bigg ) \end{aligned}$$

and

3.27 $$\begin{aligned} D\bigg (\varphi (z), \tilde{\epsilon }\bigg (1 - \frac{\epsilon }{|z - z_0|}\bigg )\bigg ) \subset \varphi (D(z,\epsilon )) \subset D\bigg (\varphi (z), \tilde{\epsilon }\bigg (1 + \frac{\epsilon }{|z - z_0|}\bigg )\bigg ), \end{aligned}$$

where

$$\begin{aligned} \tilde{\epsilon } = \frac{\epsilon }{|z - z_0|^2 - \epsilon ^2}. \end{aligned}$$

Noting that

$$\begin{aligned} 1 \pm \frac{\epsilon }{|z - z_0|} = 1 \pm g(\tilde{\epsilon }) \end{aligned}$$

where

$$\begin{aligned} g(\tilde{\epsilon }) = \frac{\sqrt{1 + 4|z - z_0|^2 \tilde{\epsilon }^2} - 1}{2|z - z_0|\tilde{\epsilon }}, \end{aligned}$$

we can write (3.27) as

3.28 $$\begin{aligned} D(\varphi (z), \tilde{\epsilon }(1 - g(\tilde{\epsilon }))) \subset \varphi (D(z,\epsilon )) \subset D(\varphi (z), \tilde{\epsilon }(1 + g(\tilde{\epsilon }))). \end{aligned}$$

The function $(\Phi h)(w) = w^{-1}h(w^{-1} + z_0)$ is the restriction to $\varphi (\Gamma )$ of a smooth function which approaches zero as $w \rightarrow \infty$. Hence there exists an $M >0$ such that $|(\Phi h)(w)| \le M$ for all $w \in \varphi (\Gamma )$. We estimate

3.29 $$\begin{aligned} \bigg | \int _{\varphi (\Gamma ) {\setminus } \varphi (D(z, \epsilon ))}&\frac{(\Phi h)(w')}{w' - \varphi (z)} dw' - \int _{\varphi (\Gamma ) {\setminus } D(\varphi (z), \tilde{\epsilon }(1 + g(\tilde{\epsilon })))} \frac{(\Phi h)(w')}{w' - \varphi (z)} dw'\bigg | \nonumber \\&= \bigg | \int _{\varphi (\Gamma ) \cap [D(\varphi (z), \tilde{\epsilon }(1 + g(\tilde{\epsilon }))) {\setminus } \varphi (D(z, \epsilon ))]} \frac{(\Phi h)(w')}{w' - \varphi (z)} dw' \bigg | \nonumber \\&\le M \int _{\varphi (\Gamma ) \cap [D(\varphi (z), \tilde{\epsilon }(1 + g(\tilde{\epsilon }))) {\setminus } D(\varphi (z), \tilde{\epsilon }(1 - g(\tilde{\epsilon })))]} \frac{|dw'|}{|w' - \varphi (z)|}. \end{aligned}$$

Being locally rectifiable, the Carleson jump contour $\varphi (\Gamma )$ has a two-sided tangent at almost every point. Hence, by Lemma 3.12, the limit of the right-hand side of (3.29) as $\epsilon \rightarrow 0$ exists and equals zero for a.e. $z \in \Gamma$. On the other hand, by Proposition 3.11, the limit

$$\begin{aligned} \lim _{\epsilon \rightarrow 0} \frac{1}{\pi i} \int _{\varphi (\Gamma ) {\setminus } D(\varphi (z), \tilde{\epsilon }(1 + g(\tilde{\epsilon })))} \frac{(\Phi h)(w')}{w' - \varphi (z)} dw' \end{aligned}$$

exists and equals $(S_{\varphi (\Gamma )}\Phi h)(\varphi (z))$ for a.e. $z \in \Gamma$. It follows that

$$\begin{aligned} \lim _{\epsilon \rightarrow 0} \frac{\varphi (z)}{\pi i} \int _{\varphi (\Gamma ) {\setminus } \varphi (D(z, \epsilon ))} \frac{(\Phi h)(w')}{w' - \varphi (z)} dw' = \varphi (z) (S_{\varphi (\Gamma )}\Phi h)(\varphi (z)) \end{aligned}$$

for a.e. $z \in \Gamma$. This completes the proof. $\square$

## Cauchy integrals over Carleson jump contours

The following two theorems generalize Theorems 2.2 and 2.3 to the case where $\Gamma$ is a general Carleson jump contour.

### Theorem 4.1

Let $\Gamma \subset \hat{\mathbb {C}}$ be a Carleson jump contour and let $D_\pm \subset \hat{\mathbb {C}}$ be the associated open sets such that $\partial D_+ = -\partial D_- = \Gamma$. Let $\mathcal {C}$ denote the Cauchy integral operator defined in (2.6).

- Let $1 \le p < \infty$. If $f \in \dot{E}^p(D_+)$, then the nontangential limits of *f*(*z*) as *z* approaches the boundary exist a.e. on $\Gamma$; if $f_+(z)$ denotes the boundary function, then $f_+ \in \dot{L}^p(\Gamma )$ and
  4.1 $$\begin{aligned} (\mathcal {C} f_+)(z) = {\left\{ \begin{array}{ll} f(z), &{} z \in D_+, \\ 0, &{} z \in D_-. \end{array}\right. } \end{aligned}$$
  If $f \in \dot{E}^p(D_-)$, then the nontangential limits of *f*(*z*) as *z* approaches the boundary exist a.e. on $\Gamma$. If $f_-(z)$ denotes the boundary function, then $f_- \in \dot{L}^p(\Gamma )$ and
  4.2 $$\begin{aligned} (\mathcal {C} f_-)(z) = {\left\{ \begin{array}{ll} 0, &{} z \in D_+, \\ - f(z), &{} z \in D_-. \end{array}\right. } \end{aligned}$$
  In particular, $f = \mathcal {C}(f_+ - f_-)$ for all $f \in \dot{E}^p(D_+ \cup D_-)$.
- Let $1< p < \infty$. Then the Cauchy singular operator $\mathcal {S}_\Gamma :\dot{L}^p(\Gamma ) \rightarrow \dot{L}^p(\Gamma )$ defined in (2.3) satisfies $\mathcal {S}_\Gamma ^2 = I$. Moreover, if $h \in \dot{L}^p(\Gamma )$, then
  4.3 $$\begin{aligned} \mathcal {C}h|_{D_+} \in \dot{E}^p(D_+), \qquad \mathcal {C}h|_{D_-} \in \dot{E}^p(D_-). \end{aligned}$$

Theorem 4.1 implies that if $\Gamma$ is a Carleson jump contour and $h \in \dot{L}^p(\Gamma )$ for some $1< p < \infty$, then the left and right nontangential boundary values of $\mathcal {C}h$, which we denote by $\mathcal {C}_+ h$ and $\mathcal {C}_- h$, lie in $\dot{L}^p(\Gamma )$. This allows us to define two linear operators $\mathcal {C}_\pm :h \mapsto \mathcal {C}_\pm h$ on $\dot{L}^p(\Gamma )$.

### Theorem 4.2

Let $1< p < \infty$ and let $\Gamma \subset \hat{\mathbb {C}}$ be a Carleson jump contour. Then $\mathcal {C}_\pm$ are bounded operators on $\dot{L}^p(\Gamma )$ with the following properties:

- The Sokhotski-Plemelj formulas
  4.4 $$\begin{aligned} \mathcal {C}_+ = \frac{1}{2}(I + \mathcal {S}_\Gamma ), \qquad \mathcal {C}_- = \frac{1}{2}(-I + \mathcal {S}_\Gamma ), \end{aligned}$$
  are valid.
- $\mathcal {C}_\pm$ are orthogonal projections on $\dot{L}^p(\Gamma )$ in the sense that
  $$\begin{aligned} \dot{L}^p(\Gamma ) = \mathcal {C}_+\dot{L}^p(\Gamma ) \oplus \mathcal {C}_-\dot{L}^p(\Gamma ) \end{aligned}$$
  and
  $$\begin{aligned} \mathcal {C}_+ - \mathcal {C}_- = \mathbb {I}, \qquad \mathcal {C}_+^2 = \mathcal {C}_+, \qquad \mathcal {C}_-^2 = -\mathcal {C}_-, \qquad \mathcal {C}_+\mathcal {C}_- = \mathcal {C}_-\mathcal {C}_+ = 0. \end{aligned}$$
- If $$h = \mathcal {C}_+h - \mathcal {C}_-h \in \dot{L}^p(\Gamma )$$, then
  4.5 $$\begin{aligned} (\mathcal {C}h)|_{D_+} = (\mathcal {C}\mathcal {C}_+h)|_{D_+} \in \dot{E}^p(D_+), \qquad (\mathcal {C}h)|_{D_-} = -(\mathcal {C}\mathcal {C}_-h)|_{D_-} \in \dot{E}^p(D_-). \end{aligned}$$
- The map $h \mapsto (\mathcal {C}h)|_{D_+}$ is a bijection $\mathcal {C}_+\dot{L}^p(\Gamma ) \rightarrow \dot{E}^p(D_+)$ with inverse $f \mapsto f_+$.
- The map $h \mapsto (\mathcal {C}h)|_{D_-}$ is a bijection $\mathcal {C}_-\dot{L}^p(\Gamma ) \rightarrow \dot{E}^p(D_-)$ with inverse $f \mapsto -f_-$.

In the special case of a jump contour $\Gamma$ consisting of a single rectifiable Jordan curve, Theorems 4.1 and 4.2 reduce to Theorems 2.2 and 2.3, respectively.

### Proof of Theorem 4.1

#### Proof of (*a*)

Suppose first that $\infty \notin \Gamma$, so that $\Gamma \subset \mathbb {C}$ is bounded. Let $f \in \dot{E}^p(D_+)$. Represent $\Gamma$ as the union of finitely many arcs each pair of which have at most endpoints in common. If $z \in \Gamma$ is not one of these finitely many endpoints, then *z* belongs to $\partial D_j^+$ for exactly one component $D_j^+$ of $D_+$. Since Theorem 2.2 implies that $f|_{D_j^+}$ has nontangential limits a.e. on $\partial D_j^+$, it follows that *f* has nontangential limits a.e. on $\Gamma$. Another application of Theorem 2.2 shows that $f_+|_{\partial D_j^+} \in L^p(\partial D_j^+)$ for each *j*. Hence $f_+ \in L^p(\Gamma ) = \dot{L}^p(\Gamma )$.

Now suppose $z \in D_{k}^+$ for some $1 \le k \le n$. Since *z* lies in the region exterior to $\partial D_j^+$ for each $j \ne k$, Theorem 2.2 yields

$$\begin{aligned} (\mathcal {C}f_+)(z) = \frac{1}{2\pi i} \int _{\partial D_k^+} \frac{f_+(z')}{z' - z}dz' + \frac{1}{2\pi i} \sum _{j \ne k} \int _{\partial D_j^+} \frac{f_+(z')}{z' - z}dz' = f(z). \end{aligned}$$

If $z \in D_-$, then *z* lies in the region exterior to $\partial D_j^+$ for every *j*, so a similar computation implies $(\mathcal {C}f_+)(z) = 0$. This proves (4.1). Similar arguments apply when $f \in \dot{E}^p(D_-)$. This proves (*a*) in the case when $\Gamma$ is bounded.

Suppose now that $\infty \in \Gamma$. Pick $z_0 \in D_-$ and let $\varphi (z) = 1/(z-z_0)$. Let $f \in \dot{E}^p(D_+)$. Then $\infty \notin \varphi (\Gamma )$ and $\varphi (\Gamma )$ is a Carleson jump contour by Proposition 3.5. Let $F(w) = w^{-1} f(\varphi ^{-1}(w))$. Then $F \in \dot{E}^p(\varphi (D_+))$ and $f \circ \varphi ^{-1} \in E^p(\varphi (D_+))$ by Proposition 3.6. Since $\varphi (\Gamma )$ is bounded, the result of the preceding paragraph implies that the nontangential boundary values of $f \circ \varphi ^{-1}$ exist a.e. on $\varphi (\Gamma )$. It follows that the nontangential boundary values of *f* exist a.e. on $\Gamma$ and $(f \circ \varphi ^{-1})_+ = f_+ \circ \varphi ^{-1}$ a.e. on $\varphi (\Gamma )$. Furthermore, since $F \in \dot{E}^p(\varphi (D_+))$, we have $F_+ \in \dot{L}^p(\varphi (\Gamma ))$, which by Lemma 3.9 implies that $f_+ \in \dot{L}^p(\Gamma )$. We also have

4.6 $$\begin{aligned} (\mathcal {C}_{\varphi (\Gamma )} F_+)(w) = {\left\{ \begin{array}{ll} F(w), &{} w \in \varphi (D_+), \\ 0, &{} w \in \varphi (D_-), \end{array}\right. } \end{aligned}$$

which in view of Lemma 3.9 yields (4.1). Similar arguments apply when $f \in \dot{E}^p(D_-)$. This proves (*a*).

#### A convergence lemma

For the proof of (*b*), we need the following lemma.

##### Lemma 4.3

Let $1 \le p < \infty$. Let $\Gamma \subset \mathbb {C}$ be a rectifiable Jordan curve and let $D_+$ and $D_-$ be the interior and exterior components of $\hat{\mathbb {C}} {\setminus } \Gamma$. Suppose $h \in L^p(\Gamma )$.

(i) If $\{f_n\}_1^\infty$ is a sequence of functions in $E^p(D_+)$ such that $f_{n+} \rightarrow h$ in $L^p(\Gamma )$, then there exists a function $f \in E^p(D_+)$ such that $f_n \rightarrow f$ uniformly on compact subsets of $D_+$ and $f_+ = h$.
(ii) If $\{f_n\}_1^\infty$ is a sequence of functions in $E^p(D_-)$ such that $f_{n-} \rightarrow h$ in $L^p(\Gamma )$, then there exists a function $f \in E^p(D_-)$ such that $f_n \rightarrow f$ uniformly on compact subsets of $D_-$ and $f_- = h$. If $\{f_n\}_1^\infty \subset \dot{E}^p(D_-)$, then $f \in \dot{E}^p(D_-)$.

##### Proof

Part (*i*) is a consequence of Theorem 17.2 in Chapter III of [27]. In order to prove (*ii*), let $\{f_n\}_1^\infty$ be a sequence of functions in $E^p(D_-)$ such that $f_{n-} \rightarrow h$ in $L^p(\Gamma )$. Let $z_0\in D_+$ and let $\varphi (z) = 1/(z-z_0)$. Then $h \circ \varphi ^{-1} \in L^p(\varphi (\Gamma ))$ and Proposition 3.6 implies that $f_n \circ \varphi ^{-1} \in E^p(\varphi (D_-))$ for each *n*. Assuming for simplicity that both $\Gamma$ and $\varphi (\Gamma )$ are oriented counterclockwise, we have $(f_n \circ \varphi ^{-1})_+ = f_{n-} \circ \varphi ^{-1}$, and so

$$\begin{aligned} \Vert (f_n \circ \varphi ^{-1})_+ - h \circ \varphi ^{-1}\Vert _{L^p(\varphi (\Gamma ))}^p&= \int _{\varphi (\Gamma )} |f_{n-}(\varphi ^{-1}(w)) - h(\varphi ^{-1}(w))|^p |dw| \\&= \int _\Gamma |f_{n-}(z) - h(z)|^p \frac{|dz|}{|z-z_0|^2}\\&\le C \Vert f_{n-} - h\Vert _{L^p(\Gamma )}^p \rightarrow 0 \quad \text {as} \quad n \rightarrow \infty . \end{aligned}$$

Hence, by (*i*), there exists a function $g \in E^p(\varphi (D_-))$ such that $f_n \circ \varphi ^{-1} \rightarrow g$ uniformly on compact subsets of $\varphi (D_-)$ and $g_+ = h \circ \varphi ^{-1}$. Letting $f = g \circ \varphi$, we infer that $f \in E^p(D_-)$, that $f_n \rightarrow f$ uniformly on compact subsets of $D_-$, and that $f_- = h$. If $\{f_n\}_1^\infty \subset \dot{E}^p(D_-)$, then each $f_n$ vanishes at $\infty$. Hence *f* vanishes at $\infty$, and so $f \in \dot{E}^p(D_-)$. $\square$

#### Proof of (*b*)

Suppose $1< p < \infty$ and $h \in \dot{L}^p(\Gamma )$. We first assume that $\infty \notin \Gamma$. Switching the orientation of $\Gamma$ if necessary, we may suppose that $\infty \in D_-$. Let $R(\Gamma )$ be the set of all rational functions with no poles on $\Gamma$. Every function $r \in R(\Gamma )$ can be written as $r = r^+ + r^-$, where $r^+$ is analytic in $D_+$, $r^-$ is analytic in $D_-$, and $r^-$ vanishes at infinity. That is, $r^+ \in \dot{E}^p(D_+)$ and $r^- \in \dot{E}^p(D_-)$. We claim that

4.7 $$\begin{aligned} \mathcal {S}_\Gamma r^+ = r^+, \qquad \mathcal {S}_\Gamma r^- = -r^-. \end{aligned}$$

Indeed, if $\Gamma$ consists of a single Carleson Jordan curve, then (4.7) follows from Lemma 6.5 of [4]. If $\Gamma$ is the union of multiple Carleson Jordan curves $\{\partial D_j^+\}_1^n$, then we write $r^- = \sum _{j=1}^n r_j^-$ where $r_j^-$ is analytic outside $D_j^+$ and $r_j^-(\infty ) = 0$ for each *j*. Let $\chi _j$ be the characteristic function of $\partial D_j^+$. Decomposing $r^+$ and $r_j^-$ into partial fractions and using that (4.7) is valid in the case when $\Gamma$ is a Carleson Jordan curve, we find

4.8 $$\begin{aligned} \chi _k \mathcal {S}_\Gamma \chi _i r^+ = {\left\{ \begin{array}{ll} \chi _k r^+, &{}\quad k = i, \\ 0, &{}\quad k \ne i, \end{array}\right. } \end{aligned}$$

and

4.9 $$\begin{aligned} \chi _k \mathcal {S}_\Gamma \chi _i r_j^- = {\left\{ \begin{array}{ll} -\chi _kr_j^-, &{}\quad k = i = j, \\ \chi _k r_j^-, &{}\quad k = i \ne j, \\ -2\chi _k r_j^-, &{}\quad k \ne i = j, \\ 0, &{}\quad k \ne i \ne j, \end{array}\right. } \end{aligned}$$

a.e. on $\Gamma$. Equation (4.9) implies

$$\begin{aligned} \mathcal {S}_\Gamma r_j^-&= \sum _{k,i = 1}^n \chi _k \mathcal {S}_\Gamma \chi _i r_j^- = \chi _j \mathcal {S}_\Gamma \chi _j r_j^- + \sum _{k = i \ne j} \chi _k \mathcal {S}_\Gamma \chi _i r_j^- + \sum _{k \ne i = j} \chi _k\mathcal {S}_\Gamma \chi _i r_j^- \\&= -\chi _jr_j^- + \sum _{k \ne j} \chi _k r_j^- - 2 \sum _{k \ne j} \chi _k r_j^- = -r_j^-, \qquad 1 \le j \le n. \end{aligned}$$

Thus $\mathcal {S}_\Gamma r^- = - r^-$. Similarly, equation (4.8) implies $\mathcal {S}_\Gamma r^+ = r^+$. This proves (4.7).

Equation (4.7) implies that $\mathcal {S}_\Gamma ^2 r = r$ for every $r \in R(\Gamma )$. Since $R(\Gamma )$ is dense in $L^p(\Gamma )$ (see Lemma 9.14 in [4]), it follows that $\mathcal {S}_\Gamma ^2h = h$ for every $h \in L^p(\Gamma )$.

To prove (4.3), we note that part (*a*) yields

4.10 $$\begin{aligned} (\mathcal {C} r^+)(z) = {\left\{ \begin{array}{ll} r^+(z), &{} z \in D_+, \\ 0, &{} z \in D_-, \end{array}\right. } \qquad (\mathcal {C} r^-)(z) = {\left\{ \begin{array}{ll} 0, &{} z \in D_+, \\ -r^-(z), &{} z \in D_-. \end{array}\right. } \end{aligned}$$

It follows that $\mathcal {C}r \in \dot{E}^p(D_+ \cup D_-)$ for every $r \in R(\Gamma )$ and that

4.11 $$\begin{aligned} \mathcal {C}_+ r = r^+, \qquad \mathcal {C}_- r = -r^-. \end{aligned}$$

Equations (4.7) and (4.11) imply

$$\begin{aligned} \frac{1}{2}(I + \mathcal {S}_\Gamma ) r = \frac{1}{2}(r^+ + r^- + r^+ -r^-) = r^+ = \mathcal {C}_+ r . \end{aligned}$$

Similarly,

$$\begin{aligned} \frac{1}{2}(-I + \mathcal {S}_\Gamma ) r = \mathcal {C}_- r. \end{aligned}$$

This shows that the Sokhotski-Plemelj formulas (4.4) are valid for all $r \in R(\Gamma )$.

Let $h \in L^p(\Gamma )$. Let $r_n$ be a sequence in $R(\Gamma )$ converging to *h* in $L^p(\Gamma )$. The boundedness of $\mathcal {S}_\Gamma$ on $L^p(\Gamma )$ implies

$$\begin{aligned} \mathcal {C}_\pm r_n = \frac{1}{2}(\pm r_n + \mathcal {S}_\Gamma r_n) \rightarrow \frac{1}{2}(\pm h + \mathcal {S}_\Gamma h) \quad \text {in} \quad L^p(\Gamma ) \end{aligned}$$

as $n \rightarrow \infty$. Hence Lemma 4.3 applied to each component of $\hat{\mathbb {C}} {\setminus } \Gamma$ implies that there exists a function $f \in \dot{E}^p(D_+ \cup D_-)$ such that $(\mathcal {C}r_n)|_{D_+ \cup D_-} \rightarrow f$ uniformly on compact subsets of $D_+ \cup D_-$ and $f_\pm = \frac{1}{2}(\pm h + \mathcal {S}_\Gamma h)$. Since $\mathcal {C}r_n \rightarrow \mathcal {C}h$ pointwise in $D_+$, we infer that $\mathcal {C}h = f \in \dot{E}^p(D_+ \cup D_-)$. This proves (4.3) in the case of $\infty \notin \Gamma$. It also follows that

$$\begin{aligned} \mathcal {C}_\pm h = f_\pm = \frac{1}{2}(\pm h + \mathcal {S}_\Gamma h), \end{aligned}$$

showing that the Sokhotski-Plemelj formulas (4.4) are valid for all $h \in L^p(\Gamma )$.

Suppose now that $\infty \in \Gamma$. Pick $z_0 \in D_-$ and let $\varphi (z) = 1/(z-z_0)$. Since $h \in \dot{L}^p(\Gamma )$, Lemma 3.9 implies that $\Phi h \in L^p(\varphi (\Gamma ))$ and

4.12 $$\begin{aligned} (\mathcal {C}_\Gamma h)(z) = (\Psi ^{-1} \mathcal {C}_{\varphi (\Gamma )} \Phi h)(z) \quad \text {for} \quad z \in \mathbb {C}{\setminus } \Gamma . \end{aligned}$$

The result of the previous paragraph implies that $\mathcal {C}_{\varphi (\Gamma )} \Phi h \in \dot{E}^p(\varphi (D_+ \cup D_-))$. Hence, in view of Proposition 3.6 and Eq. (4.12), $\mathcal {C}_\Gamma h \in \dot{E}^p(D_+ \cup D_-)$, which proves (4.3). Similarly, the identity $\mathcal {S}_\Gamma ^2 = I$ follows from the identity $\mathcal {S}_{\varphi (\Gamma )}^2 =I$ and Eq. (3.24):

$$\begin{aligned} \mathcal {S}_\Gamma ^2h = \Phi ^{-1}\mathcal {S}_{\varphi (\Gamma )}^2 \Phi h = \Phi ^{-1} \Phi h = h \quad \text {for all} \quad h \in \dot{L}^p(\Gamma ). \end{aligned}$$

This completes the proof of Theorem 4.1.

### Proof of Theorem 4.2

We already established the Sokhotski–Plemelj formulas (4.4) in the case of $\infty \notin \Gamma$ (see the proof of part (*b*) of Theorem 4.1). So suppose $\infty \in \Gamma$. Pick $z_0 \in D_-$ and let $\varphi (z) = 1/(z-z_0)$. The fact that $\infty \notin \varphi (\Gamma )$ together with the transformation properties (3.10) and (3.24) of $\mathcal {C}$ and $\mathcal {S}$ imply

$$\begin{aligned} \frac{1}{2}(\pm I + \mathcal {S}_\Gamma )h = \frac{1}{2}\Phi ^{-1}(\pm I + \mathcal {S}_{\varphi (\Gamma )})\Phi h = (\Psi ^{-1}\mathcal {C}_{\varphi (\Gamma )} \Phi h)_\pm = (\mathcal {C}_\Gamma h)_\pm . \end{aligned}$$

This completes the proof of (4.4).

The Sokhotski–Plemelj formulas (4.4) together with the fact that $\mathcal {S}_\Gamma ^2 = I$ immediately imply that $\mathcal {C}_\pm$ are bounded orthogonal projections on $\dot{L}^p(\Gamma )$.

If $h = \mathcal {C}_+h - \mathcal {C}_-h \in \dot{L}^p(\Gamma )$, then $\mathcal {C} h \in \dot{E}^p(D_+ \cup D_-)$ by (4.3). Hence equations (4.1) and (4.2) imply

$$\begin{aligned} (\mathcal {C} \mathcal {C}_+ h)(z) = {\left\{ \begin{array}{ll} (\mathcal {C}h)(z), &{} z \in D_+, \\ 0, &{} z \in D_-, \end{array}\right. } \qquad (\mathcal {C} \mathcal {C}_- h)(z) = {\left\{ \begin{array}{ll} 0, &{} z \in D_+, \\ -(\mathcal {C}h)(z), &{} z \in D_-. \end{array}\right. } \end{aligned}$$

These equations yield (4.5). The last two statements of Theorem 4.2 are easy consequences of (4.5) and Theorem 4.1. This completes the proof. $\square$

## Riemann–Hilbert problems

With Theorems 4.1 and 4.2 at our disposal, we can introduce a notion of $L^p$-RH problem for Carleson jump contours. Throughout this section, $\Gamma \subset \hat{\mathbb {C}}$ will denote a Carleson jump contour, $D_\pm \subset \hat{\mathbb {C}}$ will denote the associated open sets such that $\partial D_+ = -\partial D_- = \Gamma$, and we will assume that $1< p <\infty$. We let $D = D_+ \cup D_-$.

### Definition

Let $n \ge 1$ be an integer. Given an $n \times n$-matrix valued function $v: \Gamma \rightarrow GL(n, \mathbb {C})$, we define a *solution of the* $L^p$-*RH problem determined by* $(\Gamma , v)$ to be an $n \times n$-matrix valued function $m \in I + \dot{E}^p(D)$ such that the nontangential boundary values $m_\pm$ satisfy $m_+ = m_- v$ a.e. on $\Gamma$.

### Properties of $m_\pm$

In order to make contact with earlier works on $L^p$-RH problems on smooth contours, we show that *m* is a solution of the $L^p$-RH problem if and only if the boundary functions $m_+$ and $m_-$ satisfy the properties (RH1)-(RH2) below.

#### Proposition 5.1

Suppose $v: \Gamma \rightarrow GL(n, \mathbb {C})$. If $m \in I + \dot{E}^p(D)$ satisfies the $L^p$-RH problem determined by $(\Gamma , v)$, then the nontangential boundary values $m_\pm \in I + \dot{L}^p(\Gamma )$ satisfy the following two properties:

1. There exists a function $h \in \dot{L}^p(\Gamma )$ such that
  5.1 $$\begin{aligned} m_\pm - I = \mathcal {C}_\pm h \quad \text {in}\quad \dot{L}^p(\Gamma ). \end{aligned}$$
2. $$m_+ = m_- v$$ a.e. on $$\Gamma $$.

Conversely, if $m_\pm \in I + \dot{L}^p(\Gamma )$ are a pair of $n\times n$-matrix valued functions satisfying (RH1) and (RH2), then $m = I + \mathcal {C}(m_+ - m_-) \in I + \dot{E}^p(D)$ satisfies $L^p$-RH problem determined by $(\Gamma , v)$.

#### Proof

Theorem 4.1 implies that if $m \in I + \dot{E}^p(D)$ satisfies the $L^p$-RH problem determined by $(\Gamma , v)$, then $m_\pm \in I + \dot{L}^p(\Gamma )$ and $m = I + \mathcal {C}(m_+ - m_-)$. Thus (RH1) is satisfied with $h = m_+ - m_-$. The property (RH2) holds by definition.

Conversely, suppose $m_\pm \in I + \dot{L}^p(\Gamma )$ satisfy (RH1) and (RH2). By (RH1), $m_\pm \in I + \mathcal {C}_\pm \dot{L}^p(\Gamma )$. Thus, Theorems 4.1 and 4.2 imply that $m_\pm$ are the nontangential boundary values of the function *m* defined by $m = I + \mathcal {C}(m_+ - m_-) \in I + \dot{E}^p(D)$. It follows that *m* satisfies the $L^p$-RH problem determined by $(\Gamma , v)$. $\square$

#### Remark 5.2

In most earlier references on $L^p$-RH problems [11, 12, 16, 29], a solution of an $L^p$-RH problem is defined as a pair of functions $m_\pm \in I + L^p(\Gamma )$ satisfying (RH1)-(RH2) (or properties very similar to (RH1)-(RH2)); the associated function *m*(*z*) is then referred to as the ‘extension of $m_\pm$’. Here, in an effort to mimic the classical formulation of a RH problem as closely as possible, we have chosen to define a solution directly in terms of *m*. Proposition 5.1 shows that in the set-up provided by the spaces $\dot{L}^p(\Gamma )$ and $\dot{E}^p(D)$, the definitions in terms of *m* and $m_\pm$ are equivalent.

#### Remark 5.3

Condition (RH1) is equivalent to the condition that $m_\pm \in I + \mathcal {C}_\pm \dot{L}^p(\Gamma )$.

### Uniqueness results

We will show that the solution of the $L^p$-RH problem determined by $(\Gamma , v)$ is unique provided that $\det v = 1$ and $n \le p$.

#### Lemma 5.4

Suppose $v: \Gamma \rightarrow GL(n, \mathbb {C})$. Let $1< p < \infty$ and define *q* by $1/p + 1/q =1$. Let $m, \tilde{m} \in I + \dot{E}^p(D)$ be two solutions of the $L^p$-RH problem determined by $(\Gamma ,v)$. If $m^{-1} \in I + \dot{E}^q(D)$, then $m(z) = \tilde{m}(z)$ for all $z \in D$.

#### Proof

Suppose $m, \tilde{m} \in I + \dot{E}^p(D)$ are two solutions of the $L^p$-RH problem determined by $(\Gamma ,v)$. Suppose $m^{-1} \in I + \dot{E}^q(D)$. By Lemma 3.8,

$$\begin{aligned} \tilde{m} m^{-1} - I= & {} (\tilde{m} - I)(m^{-1} - I) + (\tilde{m} - I) + (m^{-1} - I) \in \dot{E}^1(D) + \dot{E}^p(D) \\&+ \dot{E}^q(D) \subset \dot{E}^1(D). \end{aligned}$$

Using Theorem 4.1 and the fact that $(\tilde{m} m^{-1})_+ = \tilde{m}_{-}v v^{-1} m_{-}^{-1} = (\tilde{m} m^{-1})_-$ a.e on $\Gamma$, we find

$$\begin{aligned} \tilde{m} m^{-1} - I = \mathcal {C}((\tilde{m} m^{-1} - I)_+ - (\tilde{m} m^{-1} - I)_-) = 0 \quad \text {on} \;\; D. \end{aligned}$$

It follows that $m = \tilde{m}$ on *D*. $\square$

#### Remark 5.5

The assumption in Lemma 5.4 that $m^{-1} \in I + \dot{E}^q(D)$ implies that $m_\pm$ deliver a so-called $L^p$-canonical factorization of *v*; the uniqueness of the latter is known, see [18, 24].

Suppose $v: \Gamma \rightarrow GL(2, \mathbb {C})$ satisfies $\det v = 1$ a.e. on $\Gamma$. If $m \in I + \dot{E}^2(D)$ is a solution of the $L^2$-RH problem determined by $(\Gamma ,v)$, then Lemma 3.8 shows that

$$\begin{aligned} \det m - 1 = (m_{11} - 1)(m_{22} - 1) + (m_{11} - 1) + (m_{22} - 1) - m_{12} m_{21} \in \dot{E}^1(D). \end{aligned}$$

By Theorem 4.1 and the fact that $(\det m)_+ = (\det m)_-$ a.e. on $\Gamma$, we find

$$\begin{aligned} \det m - 1 = \mathcal {C}((\det m - 1)_+ - (\det m - 1)_-) = 0 \quad \text {on} \;\; D. \end{aligned}$$

Thus,

$$\begin{aligned} m^{-1} = \begin{pmatrix} m_{22} &{} -m_{12} \\ -m_{21} &{} m_{11} \end{pmatrix} \in I + \dot{E}^2(D). \end{aligned}$$

Lemma 5.4 therefore implies that the solution of the $L^2$-RH problem determined by $(\Gamma ,v)$ is unique if it exists. This proves the special case $n = p = 2$ of the following theorem, which states that if $p \ge n$ and the $n\times n$-matrix valued jump function *v* satisfies $\det v = 1$, then the solution of the $L^p$-RH problem determined by $(\Gamma ,v)$ is unique if it exists.

Recall that the adjugate ${{\mathrm{adj}}}A$ of an $n \times n$ matrix *A* is defined by

$$\begin{aligned} ({{\mathrm{adj}}}A)_{ij} = (-1)^{i+j} m_{ji}(A), \qquad i,j = 1, \dots , n, \end{aligned}$$

where $m_{ij}(A)$ denotes the (*ij*)th minor of *A*. By Cramer’s rule, the inverse of *A* is given by $A^{-1} = {{\mathrm{adj}}}(A)/\det (A)$ whenever $\det (A) \ne 0$. We continue to assume that $1< p < \infty$.

#### Theorem 5.6

Suppose $1 \le n \le p$. Suppose $v: \Gamma \rightarrow GL(n, \mathbb {C})$ satisfies $\det v = 1$ a.e. on $\Gamma$.

- If *m* is a solution of the $L^p$-RH problem determined by $(\Gamma ,v)$, then $\det m(z) = 1$ for all $z \in D$.
- The solution of the $L^p$-RH problem determined by $(\Gamma ,v)$ is unique if it exists.

#### Proof

Let $m \in I + \dot{E}^p(D)$ be a solution of the $L^p$-RH problem determined by $(\Gamma ,v)$ for some $p \ge n$. By Lemma 3.8, if $\{f_j\}_1^k \subset \dot{E}^p(D)$ and $1 \le k \le n$, then $\Pi _{j=1}^k f_j \in \dot{E}^{p/k}(D) \subset \dot{E}^1(D)$. It follows that

$$\begin{aligned} \det m - 1 = \det (I + (m- I)) - 1 \in \dot{E}^1(D). \end{aligned}$$

Using Theorem 4.1 and the fact that $(\det m)_+ = (\det m)_-$ a.e. on $\Gamma$, we find

$$\begin{aligned} \det m - 1 = \mathcal {C}((\det m)_+ - (\det m)_-) = 0 \quad \text {on} \;\; D, \end{aligned}$$

which proves (*a*). To prove (*b*), we note that Lemma 3.8 implies

$$\begin{aligned} {{\mathrm{adj}}}m \in I + \dot{E}^{\frac{p}{n-1}}(D) \subset I + \dot{E}^{\frac{p}{p-1}}(D). \end{aligned}$$

Hence, by (*a*),

$$\begin{aligned} m^{-1} = {{\mathrm{adj}}}m \in I + \dot{E}^{\frac{p}{p-1}}(D), \end{aligned}$$

so that (*b*) follows from Lemma 5.4. $\square$

#### Remark 5.7

For a sufficiently smooth contour, the special case $n = p = 2$ of Theorem 5.6 was proved in [8, 13]. Theorem 5.6 generalizes this result to the case of a Carleson contour $\Gamma$ and any $1 \le n \le p$. As an application, we note that the case $n=3$ is relevant for the $3 \times 3$-matrix RH problem associated with the Degasperis–Procesi equation, see Fig. 1.

### A singular integral equation

Given a Banach space *X*, we let $\mathcal {B}(X)$ denote the space of bounded linear operators on *X*. Given two functions $w^\pm \in \dot{L}^p(\Gamma ) \cap L^\infty (\Gamma )$, we define the operator $\mathcal {C}_{w}: \dot{L}^p(\Gamma ) + L^\infty (\Gamma ) \rightarrow \dot{L}^p(\Gamma )$ by

$$\begin{aligned} \mathcal {C}_{w}(f) = \mathcal {C}_+(f w^-) + \mathcal {C}_-(f w^+). \end{aligned}$$

We fix a point $z_0 \in \mathbb {C}{\setminus } \Gamma$ and let $\Vert \cdot \Vert _{\dot{L}^p(\Gamma )}$ denote the associated norm on $\dot{L}^p(\Gamma )$ defined in (3.12). The estimate

$$\begin{aligned} \Vert \mathcal {C}_wf\Vert _{\dot{L}^p(\Gamma )}&= \Vert \mathcal {C}_+(f w^-) + \mathcal {C}_-(f w^+)\Vert _{\dot{L}^p(\Gamma )} \\&\le C \Vert f\Vert _{\dot{L}^p(\Gamma )} \max \big \{\Vert w^+\Vert _{L^\infty (\Gamma )}, \Vert w^-\Vert _{L^\infty (\Gamma )} \big \} \quad \text {for} \quad f \in \dot{L}^p(\Gamma ), \end{aligned}$$

where $C = \max \{\Vert \mathcal {C}_+\Vert _{\mathcal {B}(\dot{L}^p(\Gamma ))}, \Vert \mathcal {C}_-\Vert _{\mathcal {B}(\dot{L}^p(\Gamma ))}\} < \infty$, implies that

5.2 $$\begin{aligned} \Vert \mathcal {C}_w\Vert _{\mathcal {B}(\dot{L}^p(\Gamma ))} \le C \max \big \{\Vert w^+\Vert _{L^\infty (\Gamma )}, \Vert w^-\Vert _{L^\infty (\Gamma )} \big \}. \end{aligned}$$

The next proposition shows that if $v = (v^-)^{-1}v^+$ and $w^\pm = \pm v^\pm \mp I$ then the $L^p$-RH problem determined by $(\Gamma , v)$ is equivalent to the following singular integral equation for $\mu \in I + \dot{L}^p(\Gamma )$ cf. [2]:

5.3 $$\begin{aligned} \mu - I = \mathcal {C}_w(\mu ) \quad \text {in}\quad \dot{L}^p(\Gamma ). \end{aligned}$$

#### Proposition 5.8

Given $v^\pm : \Gamma \rightarrow GL(n, \mathbb {C})$, let $v = (v^-)^{-1}v^+$, $w^+ = v^+ - I$, and $w^- = I - v^-$. Suppose $v^\pm , (v^\pm )^{-1} \in I + \dot{L}^p(\Gamma ) \cap L^\infty (\Gamma )$. If $m \in I + \dot{E}^p(D)$ satisfies the $L^p$-RH problem determined by $(\Gamma , v)$, then $\mu = m_+ (v^+)^{-1} = m_- (v^-)^{-1} \in I + \dot{L}^p(\Gamma )$ satisfies (5.3). Conversely, if $\mu \in I + \dot{L}^p(\Gamma )$ satisfies (5.3) , then $m = I + \mathcal {C}(\mu (w^+ + w^-)) \in I + \dot{E}^p(D)$ satisfies the $L^p$-RH problem determined by $(\Gamma , v)$.

#### Proof

Suppose $m \in I + \dot{E}^p(D)$ satisfies the $L^p$-RH problem determined by $(\Gamma , v)$ and let $\mu = m_+ (v^+)^{-1} = m_- (v^-)^{-1}$. By Theorem 4.1, $m_\pm = I + \dot{L}^p(\Gamma )$ and hence $\mu \in I + \dot{L}^p(\Gamma )$. Moreover, by Theorem 4.2,

$$\begin{aligned} \mathcal {C}_w\mu&= \mathcal {C}_+(\mu (I - v^-) ) - \mathcal {C}_-(\mu (I - v^+)) \\&= \mathcal {C}_+(\mu - I + I - m_-) - \mathcal {C}_-(\mu - I + I - m^+)) \\&= (\mathcal {C}_+ - \mathcal {C}_-)(\mu - I) = \mu - I. \end{aligned}$$

Conversely, suppose $\mu \in I + \dot{L}^p(\Gamma )$ satisfies (5.3). The assumption $v^\pm \in I + \dot{L}^p(\Gamma ) \cap L^\infty (\Gamma )$ implies that $\mu w^\pm \in \dot{L}^p(\Gamma )$. Hence $m = I + \mathcal {C} (\mu (w^+ + w^-)) \in I + \dot{E}^p(D)$ and

5.4a $$\begin{aligned}&m_+ = I + (\mathcal {C}_+ - \mathcal {C}_-)(\mu w^+) + \mathcal {C}_w\mu = \mu (w^+ + I) = \mu v^+, \end{aligned}$$

5.4b $$\begin{aligned}&m_- = I - (\mathcal {C}_+ - \mathcal {C}_-)(\mu w^-) + \mathcal {C}_w\mu = \mu (I - w^-) = \mu v^-. \end{aligned}$$

It follows that $m_+ = m_- v$ a.e. on $\Gamma$. $\square$

### Fredholm properties

Given $v: \Gamma \rightarrow GL(n, \mathbb {C})$, we define a *solution of the homogeneous* $L^p$-*RH problem determined by* $(\Gamma , v)$ to be an $n \times n$-matrix valued function $m \in \dot{E}^p(D)$ such that $m_+ = m_- v$ a.e. on $\Gamma$.

#### Lemma 5.9

Given $v^\pm : \Gamma \rightarrow GL(n, \mathbb {C})$, let $v = (v^-)^{-1}v^+$, $w^+ = v^+ - I$, and $w^- = I - v^-$. Suppose $v^\pm , (v^\pm )^{-1} \in I + \dot{L}^p(\Gamma ) \cap L^\infty (\Gamma )$. Then the implications

$$\begin{aligned} (a) \implies (b) \implies (c) \implies (d) \end{aligned}$$

are valid for the following statements:

- The map $I - \mathcal {C}_w: \dot{L}^p(\Gamma ) \rightarrow \dot{L}^p(\Gamma )$ is bijective.
- The $L^p$-RH problem determined by $(\Gamma , v)$ has a unique solution.
- The homogeneous $L^p$-RH problem determined by $(\Gamma , v)$ has only the zero solution.
- The map $I - \mathcal {C}_w: \dot{L}^p(\Gamma ) \rightarrow \dot{L}^p(\Gamma )$ is injective.

#### Proof

$(a) \implies (b)$ Suppose $I - \mathcal {C}_w:\dot{L}^p(\Gamma ) \rightarrow \dot{L}^p(\Gamma )$ is a bijection. Then $\mu = I + (I - \mathcal {C}_w)^{-1}\mathcal {C}_wI \in I + \dot{L}^p(\Gamma )$ is a solution of (5.3). Hence, by Proposition 5.8, $m = I + \mathcal {C}(\mu (w^+ + w^-)) \in I + \dot{E}^p(D)$ satisfies the $L^p$-RH problem determined by $(\Gamma , v)$. Moreover, by (5.4), $m_\pm = \mu v^\pm$. If $\tilde{m} \in I + \dot{E}^p(D)$ is another solution of this RH problem, then Proposition 5.8 implies that $\tilde{\mu } = \tilde{m}_\pm (v^\pm )^{-1} \in I + \dot{L}^p(\Gamma )$ is a solution of (5.3). But then $\tilde{\mu } = I + (I - \mathcal {C}_w)^{-1}\mathcal {C}_wI = \mu$, so that $\tilde{m}_\pm = m_\pm$ a.e. on $\Gamma$. Theorem 4.1 now yields

$$\begin{aligned} m = I + \mathcal {C}(m_+ - m_-) = I + \mathcal {C}(\tilde{m}_+ - \tilde{m}_-) = \tilde{m} \quad \text {in} \;\; \dot{E}^p(D), \end{aligned}$$

showing that the solution is unique.

$(b) \implies (c)$ Let $m \in I + \dot{E}^p(D)$ be the unique solution of the $L^p$-RH problem determined by $(\Gamma , v)$ and suppose $\tilde{m} \in \dot{E}^p(D)$ satisfies the homogeneous RH problem determined by $(\Gamma , v)$. By Proposition 5.8, $\mu = m_- (v^-)^{-1}$ satisfies Eq. (5.3). By Theorem 4.2,

$$\begin{aligned} (I - \mathcal {C}_w)(\tilde{m}_-(v^-)^{-1})&= \tilde{m}_-(v^-)^{-1} - \mathcal {C}_+(\tilde{m}_- (v^-)^{-1}(I - v^-)) \\&\quad - \mathcal {C}_-(\tilde{m}_- (v^-)^{-1} (v^+ - I)) \\&= \tilde{m}_-(v^-)^{-1} - \mathcal {C}_+(\tilde{m}_- (v^-)^{-1} - \tilde{m}_-)\\&\quad - \mathcal {C}_-(\tilde{m}_+ - \tilde{m}_- (v^-)^{-1}) = 0. \end{aligned}$$

Hence $\mu = (m_- + \tilde{m}_-)(v^-)^{-1}$ also satisfies Eq. (5.3). By Proposition 5.8 and uniqueness of *m*, we conclude that

$$\begin{aligned} m = I + \mathcal {C}(m_-(v^-)^{-1} (w^+ + w^-)) = I + \mathcal {C}((m_- + \tilde{m}_-) (v^-)^{-1} (w^+ + w^-)). \end{aligned}$$

But then $\mathcal {C}_\pm (\tilde{m}_- (v^-)^{-1} (w^+ + w^-)) = 0$, and so

$$\begin{aligned} \tilde{m}_+ - \tilde{m}_- = \tilde{m}_- (v^-)^{-1} (w^+ + w^-) = (\mathcal {C}_+ - \mathcal {C}_-)(\tilde{m}_- (v^-)^{-1} (w^+ + w^-)) = 0 \end{aligned}$$

a.e. on $\Gamma$. Thus, by Theorem 4.1, $\tilde{m} = \mathcal {C}(\tilde{m}_+ - \tilde{m}_-) = 0$.

$(c) \implies (d)$ Suppose the homogeneous $L^p$-RH problem determined by $(\Gamma , v)$ has only the zero solution. Suppose $h \in \dot{L}^p(\Gamma )$ satisfies $(I - \mathcal {C}_w)h = 0$. Let $m = \mathcal {C}(h (w^+ + w^-)) \in \dot{E}^p(D)$. Since

$$\begin{aligned} m_+= & {} \mathcal {C}_+ (h (w^+ + w^-)) = \mathcal {C}_+ (h w^+) + h - \mathcal {C}_- (h w^+) = h w^+ + h = h v^+,\\ m_-= & {} \mathcal {C}_- (h (w^+ + w^-)) = h - \mathcal {C}_+ (h w^-) + \mathcal {C}_- (h w^-) = h - hw^- = hv^-, \end{aligned}$$

it follows that $m_+ = m_-v$ a.e. on $\Gamma$. Hence $m = 0$ by uniqueness of the solution of the homogeneous problem. Thus $h = m_-(v^-)^{-1} = 0$, showing that $(I - \mathcal {C}_w)$ is injective. $\square$

Let $C(\Gamma )$ denote the set of restrictions to $\Gamma$ of continuous functions $\hat{\mathbb {C}} \rightarrow \mathbb {C}$. If $\Gamma \subset \hat{\mathbb {C}}$ is given the subspace topology, Tietze’s extension theorem implies that $C(\Gamma )$ coincides with the set of continuous functions $\Gamma \rightarrow \mathbb {C}$. We will show that if $w^\pm \in C(\Gamma )$ then the operator $I - \mathcal {C}_w$ is Fredholm. If, in addition, $w^\pm$ are nilpotent, the Fredholm index of this operator is zero, so that all four statements (*a*)-(*d*) of Lemma 5.9 are equivalent.

For a Banach space *X*, let $\mathcal {K}(X) \subset \mathcal {B}(X)$ denote the set of compact operators on *X*. The set of Fredholm operators $\mathcal {F}(X)$ is open in $\mathcal {B}(X)$ and the index map ${{\mathrm{Ind}}}:\mathcal {F}(X) \rightarrow \mathbb {Z}$ is constant on the connected components of $\mathcal {F}(X)$. If $X = \dot{L}^p(\Gamma )$, we define $\mathcal {B}(X)$, $\mathcal {K}(X)$, and $\mathcal {F}(X)$ as the set of bounded, compact, and Fredholm operators on $L^p(\Gamma , w)$ where $w(z) = |z - z_0|^{1 - \frac{2}{p}}$ and $z_0$ is any point of $\mathbb {C}{\setminus } \Gamma$.

Given $w^\pm , \tilde{w}^\pm \in L^\infty (\Gamma )$ such that $\tilde{w}^+ = (w^+ + I)^{-1} - I$ and $\tilde{w}^- = I - (I - w^-)^{-1}$, we define $T_w, T_{\tilde{w}}:\dot{L}^p(\Gamma ) \rightarrow \dot{L}^p(\Gamma )$ by

5.5a $$\begin{aligned}&T_w = \mathcal {C}_+ \mathcal {R}_{\tilde{w}^-} \mathcal {C}_- \mathcal {R}_{w^+ + w^-} + \mathcal {C}_- \mathcal {R}_{\tilde{w}^+} \mathcal {C}_+ \mathcal {R}_{w^+ + w^-}, \end{aligned}$$

5.5b $$\begin{aligned}&T_{\tilde{w}} = \mathcal {C}_+ \mathcal {R}_{w^-} \mathcal {C}_- \mathcal {R}_{\tilde{w}^+ + \tilde{w}^-} + \mathcal {C}_- \mathcal {R}_{w^+} \mathcal {C}_+ \mathcal {R}_{\tilde{w}^+ + \tilde{w}^-}, \end{aligned}$$

where the right multiplication operator $\mathcal {R}_g$ is defined for functions *g*(*z*) and *h*(*z*) by

$$\begin{aligned} (\mathcal {R}_g h)(z)= h(z) g(z). \end{aligned}$$

#### Theorem 5.10

Given $v^\pm : \Gamma \rightarrow GL(n, \mathbb {C})$, let $v = (v^-)^{-1}v^+$, $w^+ = v^+ - I$, and $w^- = I - v^-$. Suppose $v^\pm , (v^\pm )^{-1} \in I + \dot{L}^p(\Gamma ) \cap L^\infty (\Gamma )$ and $v^\pm \in C(\Gamma )$.

- The operator $I - \mathcal {C}_w:\dot{L}^p(\Gamma ) \rightarrow \dot{L}^p(\Gamma )$ is Fredholm.
- If $w^\pm$ are nilpotent matrices, then $I - \mathcal {C}_w$ has Fredholm index zero; in this case, each of the four statements (*a*)-(*d*) of Lemma 5.9 implies the other three.

#### Proof

Since $\Gamma \subset \hat{\mathbb {C}}$ is compact, there exists a *c* such that $|\det v^\pm | \ge c > 0$ on $\Gamma$. Thus $(v^\pm )^{-1} \in C(\Gamma )$. Let $\tilde{w}^+ = (v^+)^{-1} - I$ and $\tilde{w}^- = I - (v^-)^{-1}$. Then $\mathcal {C}_{w}$ and $\mathcal {C}_{\tilde{w}}$ are bounded $\dot{L}^p(\Gamma ) \rightarrow \dot{L}^p(\Gamma )$.

Assume first that $\infty \notin \Gamma$.

*Step 1.* We will show that $T_w$ and $T_{\tilde{w}}$ defined by (5.5) are compact operators on $L^p(\Gamma )$. By Mergelyan’s rational approximation theorem (see p. 119 of [17]), $R(\Gamma )$ is dense in $C(\Gamma )$ equipped with the $L^\infty$-norm. Let $\{w_n^\pm \}_1^\infty \subset R(\Gamma )$ be sequences such that $\lim _{n\rightarrow \infty } \Vert w^\pm - w_n^\pm \Vert _{L^\infty (\Gamma )} = 0$. Since

$$\begin{aligned} ((\mathcal {R}_{w_n^+} \mathcal {S}_\Gamma - \mathcal {S}_\Gamma \mathcal {R}_{w_n^+})h)(z) = \frac{1}{\pi i} \int _{\Gamma } h(z') \frac{w_n^+(z) - w_n^+(z')}{z' - z} dz', \qquad z \in \Gamma , \end{aligned}$$

the operators $\mathcal {R}_{w_n^+} \mathcal {S}_\Gamma - \mathcal {S}_\Gamma \mathcal {R}_{w_n^+}$ are integral operators with continuous kernels. A standard argument based on Ascoli’s theorem implies that they are compact $L^p(\Gamma ) \rightarrow C(\Gamma )$; hence they are also compact $L^p(\Gamma ) \rightarrow L^p(\Gamma )$. Since

$$\begin{aligned}&\Vert (\mathcal {R}_{w^+} \mathcal {S}_\Gamma - \mathcal {S}_\Gamma \mathcal {R}_{w^+}) - (\mathcal {R}_{w_n^+} \mathcal {S}_\Gamma - \mathcal {S}_\Gamma \mathcal {R}_{w_n^+})\Vert _{\mathcal {B}(L^p(\Gamma ))}\\&\quad \le 2 \Vert w^+ - w_n^+\Vert _{L^\infty (\Gamma )} \Vert \mathcal {S}_\Gamma \Vert _{\mathcal {B}(L^p(\Gamma ))} \rightarrow 0 \end{aligned}$$

as $n \rightarrow \infty$, it follows that

$$\begin{aligned} \mathcal {R}_{w^+} \mathcal {S}_\Gamma - \mathcal {S}_\Gamma \mathcal {R}_{w^+} = 2(\mathcal {R}_{w^+} \mathcal {C}_+ - \mathcal {C}_+ \mathcal {R}_{w^+}) \end{aligned}$$

is compact. Since the compact operators form a two-sided ideal, we find that

$$\begin{aligned} \mathcal {C}_-(\mathcal {R}_{w^+} \mathcal {C}_+ - \mathcal {C}_+ \mathcal {R}_{w}^+)\mathcal {R}_{\tilde{w}^+ + \tilde{w}^-} = \mathcal {C}_- \mathcal {R}_{w^+} \mathcal {C}_+ \mathcal {R}_{\tilde{w}^+ + \tilde{w}^-} \end{aligned}$$

is a compact operator on $L^p(\Gamma )$. Similar arguments apply to the other terms in (5.5). This shows that $T_{w}$ and $T_{\tilde{w}}$ are compact on $L^p(\Gamma )$.

*Step 2.* We will show that $I - \mathcal {C}_w$ is Fredholm on $\dot{L}^p(\Gamma )$. Let $h \in L^p(\Gamma )$. Then

$$\begin{aligned} \mathcal {C}_{\tilde{w}}\mathcal {C}_w h&=\mathcal {C}_+((\mathcal {C}_+(hw^-) + \mathcal {C}_-(hw^+))\tilde{w}^-) + \mathcal {C}_-((\mathcal {C}_+(hw^-) + \mathcal {C}_-(hw^+))\tilde{w}^+) \\&=\mathcal {C}_+((hw^- + \mathcal {C}_-(hw^-) + \mathcal {C}_-(hw^+))\tilde{w}^-) + \mathcal {C}_-((\mathcal {C}_+(hw^-) - hw^+ \\&\quad + \mathcal {C}_+(hw^+))\tilde{w}^+) \\&=T_wh + \mathcal {C}_+(hw^-\tilde{w}^-) - \mathcal {C}_-(hw^+\tilde{w}^+). \end{aligned}$$

In view of the identities $w^+ \tilde{w}^+ = - w^+ - \tilde{w}^+$ and $w^- \tilde{w}^- = w^- + \tilde{w}^-$, the right-hand side equals $T_wh + \mathcal {C}_wh + \mathcal {C}_{\tilde{w}} h$. Hence

$$\begin{aligned} I + T_w = (I - \mathcal {C}_{\tilde{w}})(I - \mathcal {C}_w). \end{aligned}$$

Interchanging *w* and $\tilde{w}$ in the above argument, we find

$$\begin{aligned} I + T_{\tilde{w}} = (I - \mathcal {C}_w)(I - \mathcal {C}_{\tilde{w}}). \end{aligned}$$

It follows that $I - \mathcal {C}_w$ is invertible modulo compact operators; hence $I - \mathcal {C}_w$ is Fredholm on $L^p(\Gamma )$. Since the norms of $L^p(\Gamma )$ and $\dot{L}^p(\Gamma )$ are equivalent when $\Gamma$ is bounded, this proves (*a*) in the case of $\infty \notin \Gamma$.

*Step 3.* The map $t \mapsto I - \mathcal {C}_{tw}$ is continuous $[0,1] \rightarrow \mathcal {B}(L^p(\Gamma ))$ because

$$\begin{aligned} \Vert \mathcal {C}_{tw} - \mathcal {C}_{sw} \Vert _{\mathcal {B}(L^p(\Gamma ))} = |t - s| \Vert \mathcal {C}_{w} \Vert _{\mathcal {B}(L^p(\Gamma ))}, \qquad t,s \in [0,1]. \end{aligned}$$

If $w^\pm$ are nilpotent, then $tw^\pm \in C(\Gamma )$ and $\det (tw^+ + I) = \det (I - tw^-) = 1$, thus the operator $I - \mathcal {C}_{tw}$ is Fredholm on $L^p(\Gamma )$ for $t \in [0,1]$ by Step 2. Since the Fredholm index is constant on connected components, this proves (*b*) in the case of $\infty \notin \Gamma$.

*Step 4.* Suppose now that $\infty \in \Gamma$. Pick $z_0 \in D_-$. Let $\Phi :\dot{L}^p(\Gamma ) \rightarrow L^p(\varphi (\Gamma ))$ be the bijection defined in (3.9). Equipping $\dot{L}^p(\Gamma )$ with the norm (3.12), $\Phi$ is an isometry by Lemma 3.9. Let $\mathcal {C} = \mathcal {C}_\Gamma$ and $\tilde{\mathcal {C}} = \mathcal {C}_{\varphi (\Gamma )}$ denote the Cauchy operators associated with the contours $\Gamma$ and $\varphi (\Gamma )$, respectively. Using (3.10), we find

5.6 $$\begin{aligned} I - \mathcal {C}_w&= I - \mathcal {C}_+R_{w^-} - \mathcal {C}_-R_{w^+} = \Phi ^{-1}(I - \Phi \mathcal {C}_+ \Phi ^{-1} \Phi R_{w^-} \Phi ^{-1}\nonumber \\&\quad - \Phi \mathcal {C}_- \Phi ^{-1} \Phi R_{w^+} \Phi ^{-1}) \Phi \nonumber \\&= \Phi ^{-1}(I - \tilde{\mathcal {C}}_+ R_{w^- \circ \varphi ^{-1}} - \tilde{\mathcal {C}}_- R_{w^+ \circ \varphi ^{-1}}) \Phi = \Phi ^{-1}(I - \tilde{\mathcal {C}}_{w \circ \varphi ^{-1}}) \Phi . \end{aligned}$$

Since $v^\pm \circ \varphi ^{-1}: \varphi (\Gamma ) \rightarrow GL(n, \mathbb {C})$ satisfy $v^\pm \circ \varphi ^{-1} \in C(\varphi (\Gamma ))$ as well as

$$\begin{aligned} v^\pm \circ \varphi ^{-1}, (v^\pm )^{-1} \circ \varphi ^{-1} \in I + L^p(\varphi (\Gamma )) \cap L^\infty (\varphi (\Gamma )), \end{aligned}$$

Step 2 implies that the operator $I - \tilde{\mathcal {C}}_{w \circ \varphi ^{-1}} \in \mathcal {B}(L^p(\varphi (\Gamma )))$ is Fredholm. Since $\Phi$ is an isometry, Eq. (5.6) implies that $I - \mathcal {C}_w \in \mathcal {B}(\dot{L}^p(\Gamma ))$ is also Fredholm of the same index. $\square$

### Reversal of subcontours

It is sometimes convenient to consider RH problems with jumps across contours which are not Carleson jump contours but which can be turned into Carleson jump contours by reorienting an appropriate subcontour. We make the following definition: If $\tilde{\Gamma }$ denotes the Carleson jump contour $\Gamma$ with the orientation reversed on a subset $\Gamma _0 \subset \Gamma$ and $\tilde{v}$ is defined by

$$\begin{aligned} \tilde{v} = {\left\{ \begin{array}{ll} v &{}\quad \text {on }\quad \Gamma {\setminus } \Gamma _0, \\ v^{-1} &{}\quad \text {on }\quad \Gamma _0, \end{array}\right. } \end{aligned}$$

then we say that $m \in I + \dot{E}^p(D)$ satisfies the $L^p$-RH problem determined by $(\tilde{\Gamma }, \tilde{v})$ if and only if *m* satisfies the $L^p$-RH problem determined by $(\Gamma , v)$.

### Contour deformations

Many applications of RH problems rely on arguments involving contour deformations. For example, in the nonlinear steepest descent method of [10], the jump contour is deformed in such a way that $w = v - I$ is exponentially small away from a finite number of critical points. Theorem 5.12 below gives conditions under which the deformed RH problem is equivalent to the original one.

#### Lemma 5.11

Let *D* be the union of any number of components of $\hat{\mathbb {C}} {\setminus } \Gamma$, where $\Gamma$ is a Carleson jump contour. Let $E^\infty (D)$ denote the space of bounded analytic functions in *D*. If $f \in \dot{E}^p(D)$ and $g \in E^\infty (D)$, then $fg \in \dot{E}^p(D)$.

#### Proof

The result is immediate when $\infty \notin \Gamma$. The case of $\infty \in \Gamma$ can be reduced to the case of $\infty \notin \Gamma$ by means of Proposition 3.6. $\square$

**Fig. 5:**
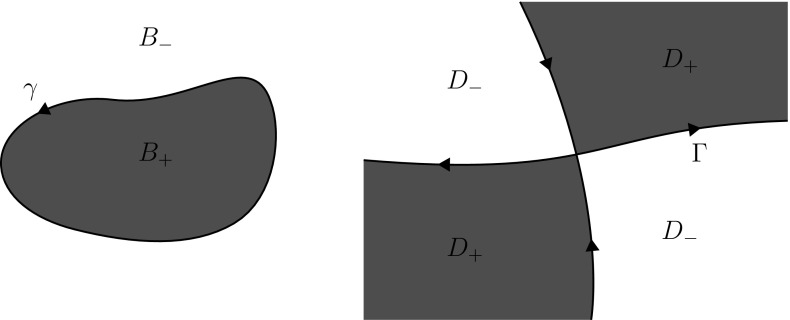
The contours $\gamma$ and $\Gamma$

**Fig. 6:**
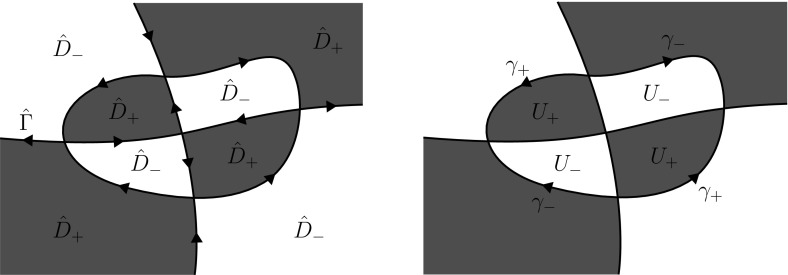
The contours $\hat{\Gamma } = \Gamma \cup \gamma$ and $\gamma _\pm$

Let $\hat{\Gamma } = \Gamma \cup \gamma$ denote the union of the Carleson jump contour $\Gamma$ and a curve $\gamma \in \mathcal {J}$, see Figs. 5 and 6. Suppose that, reversing the orientation on a subcontour if necessary, $\hat{\Gamma }$ is a Carleson jump contour. To be definite, we henceforth fix an orientation on the contour $\hat{\Gamma }$ which turns it into a Carleson jump contour, and we endow the contours $\Gamma$ and $\gamma$ with the orientations they inherit as subsets of $\hat{\Gamma }$. Then $\Gamma$ is a Carleson jump contour up to reorientation; we define a solution of the $L^p$-RH problem determined by $(\Gamma , v)$ as in Sect. 5.6.

Let $B_+$ and $B_-$ denote the two components of $\hat{\mathbb {C}} {\setminus } \gamma$. Without loss of generality, we may assume that $\infty \in \bar{B}_-$. Let $\hat{D}_\pm$ be the open sets such that $\hat{\mathbb {C}} {\setminus } \hat{\Gamma } = \hat{D}_+ \cup \hat{D}_-$ and $\partial \hat{D}_+ = - \partial \hat{D}_- = \hat{\Gamma }$. Let $U_\pm = \hat{D}_\pm \cap B_+$. Let $\hat{D} = \hat{D}_+ \cup \hat{D}_-$ and $U = U_+ \cup U_-$. Let $\gamma _+$ and $\gamma _-$ be the parts of $\gamma$ that belong to the boundary of $U_+$ and $U_-$, respectively. The orientations of $\gamma _\pm$ are such that $B_+$ lies to the left of $\gamma _+$, whereas $B_+$ lies to the right of $\gamma _-$.

#### Theorem 5.12

Let $1< p < \infty$ and let $n \ge 1$ be an integer. Suppose $v: \Gamma \rightarrow GL(n, \mathbb {C})$. Suppose $m_0:U \rightarrow GL(n,\mathbb {C})$ satisfies

$$\begin{aligned} m_0, m_0^{-1} \in I + \dot{E}^p(U) \cap E^\infty (U). \end{aligned}$$

Define $$\hat{v}:\hat{\Gamma } \rightarrow GL(n, \mathbb {C})$$ by

$$\begin{aligned} \hat{v} = {\left\{ \begin{array}{ll} m_{0-} v m_{0+}^{-1} &{}\quad \text {on} \quad \Gamma \cap B_+, \\ m_{0+}^{-1} &{} \quad \text {on} \quad \gamma _+, \\ m_{0-} &{}\quad \text {on} \quad \gamma _-, \\ v &{}\quad \text {on} \quad \Gamma \cap B_-. \end{array}\right. } \end{aligned}$$

Then the $L^p$-RH problems determined by $(\Gamma ,v)$ and $(\hat{\Gamma }, \hat{v})$ are equivalent in the following sense: If $m \in I + \dot{E}^p(D)$ satisfies the $L^p$-RH problem determined by $(\Gamma ,v)$, then the function $\hat{m}(z)$ defined for $z \in \hat{D}$ by

5.7 $$\begin{aligned} \hat{m} = {\left\{ \begin{array}{ll} mm_0^{-1} &{}\quad \text {on} \quad \hat{D} \cap B_+,\\ m &{}\quad \text {on} \quad \hat{D} \cap B_-, \end{array}\right. } \end{aligned}$$

satisfies the $L^p$-RH problem determined by $(\hat{\Gamma }, \hat{v})$.

Conversely, if $\hat{m} \in I + \dot{E}^p(\hat{D})$ satisfies the $L^p$-RH problem determined by $(\hat{\Gamma }, \hat{v})$, then the function *m*(*z*) defined for $z \in \hat{D}$ by

5.8 $$\begin{aligned} m = {\left\{ \begin{array}{ll} \hat{m}m_0 &{}\quad \text {on} \quad \hat{D} \cap B_+,\\ \hat{m} &{}\quad \text {on} \quad \hat{D} \cap B_-, \end{array}\right. } \end{aligned}$$

and extended to $D \cap \gamma$ by continuity, satisfies the $L^p$-RH problem determined by $(\Gamma , v)$.

#### Proof

Suppose $$m \in I + \dot{E}^p(D)$$ satisfies the $$L^p$$-RH problem determined by $$(\Gamma ,v)$$. Define $$\hat{m}(z)$$ for $$z \in \hat{D}$$ by (5.7). Using the identity $$mm_0^{-1} = (m-I)m_0^{-1} + m_0^{-1} $$ and Lemma 5.11, we find that $$\hat{m} \in I + \dot{E}^p(\hat{D})$$. The nontangential boundary values $$\hat{m}_\pm \in I + \dot{L}^p(\hat{\Gamma })$$ satisfy

$$\begin{aligned} \hat{m}_\pm = {\left\{ \begin{array}{ll} m_\pm m_{0\pm }^{-1} &{}\quad \text {on} \quad \hat{\Gamma } \cap B_+,\\ m_\pm , &{}\quad \text {on} \quad \hat{\Gamma } \cap B_-. \end{array}\right. } \end{aligned}$$

Moreover, $\hat{m}_+ = m_+ m_{0+}^{-1}$ and $\hat{m}_- = m_-$ on $\gamma _+$, while $\hat{m}_+ = m_+$ and $\hat{m}_- = m_-m_{0-}^{-1}$ on $\gamma _-$. It follows that $\hat{m}_+ = \hat{m}_- \hat{v}$ a.e. on $\hat{\Gamma }$. Hence $\hat{m}$ satisfies the $L^p$-RH problem determined by $(\hat{\Gamma }, \hat{v})$.

Conversely, suppose $\hat{m} \in I + \dot{E}^p(\hat{D})$ satisfies the $L^p$-RH problem determined by $(\hat{\Gamma }, \hat{v})$ and define *m*(*z*) for $z \in \mathbb {C}{\setminus } \hat{\Gamma }$ by (5.8). By Lemma 5.11, $m \in I + \dot{E}^p(\hat{D})$. The nontangential boundary values $m_\pm \in I + \dot{L}^p(\hat{\Gamma })$ satisfy

$$\begin{aligned} m_\pm = {\left\{ \begin{array}{ll} \hat{m}_\pm m_{0\pm } &{}\quad \text {on} \quad \hat{\Gamma } \cap B_+,\\ \hat{m}_\pm , &{}\quad \text {on} \quad \hat{\Gamma } \cap B_-. \end{array}\right. } \end{aligned}$$

Moreover, $m_+ = \hat{m}_+ m_{0+}$ and $m_- = \hat{m}_-$ on $\gamma _+$, while $m_+ = \hat{m}_+$ and $m_- = \hat{m}_-m_{0-}$ on $\gamma _-$. It follows that $m_+ = m_- v$ a.e. on $\Gamma$ and that $m_+ = m_-$ a.e. on $\gamma$. Using Theorem 4.1 and the fact that $m_+ = m_-$ a.e. on $\gamma$, we find

5.9 $$\begin{aligned} m(z) - I = (\mathcal {C}_{\hat{\Gamma }}(m_+ - m_-))(z) = (\mathcal {C}_{\Gamma }(m_+ - m_-))(z), \qquad z \in \hat{D}. \end{aligned}$$

Since the right-hand side belongs to $\dot{E}^p(D)$ by part (*b*) of Theorem 4.1, defining *m*(*z*) for $z \in \gamma$ by $m(z) = m_+(z) = m_-(z)$, we have $m \in I + \dot{E}^p(D)$ and equation (5.9) becomes valid for all $z \in D$. It follows that *m* satisfies the $L^p$-RH problem determined by $(\Gamma , v)$. $\square$

## Conclusions

We have taken a first few steps toward developing a theory of $L^p$-Riemann–Hilbert problems for a class of jump contours of very low regularity. More precisely, we have considered jump contours $\Gamma$ which are the union of a finite number of possibly unbounded simple Carleson curves. Several results well-known from the case of smooth contours have been shown to generalize to this more general setting. Our definition of a solution of the $L^p$-RH problem has been novel in that it has been given directly in terms of *m*(*z*) using appropriate Smirnoff classes (and not in terms of $m_\pm$ as in [11, 12, 16, 29]). Moreover, we have established uniqueness of the $L^p$-RH problem for $n\times n$ matrices for any $1 \le n \le p$ (see Theorem 5.6; for $n = p = 2$ this result was proved in [8, 13] for sufficiently smooth contours). Overall it has been demonstrated that the theory of $L^p$-RH problems extends virtually unimpeded to the setting of Carleson jump contours.

On the other hand, it is natural to expect the class of Carleson contours to be the largest class of contours for which a clean RH theory exists. Indeed, the Cauchy singular operator $\mathcal {S}_\Gamma$, which is essential in the RH formalism, is known to be bounded on $L^p(\Gamma )$, $1< p < \infty$, if and only if $\Gamma$ is a Carleson curve [4].

The presented results can be used to determine rigorously the long-time asymptotics of solutions of integrable evolution equations via the method of nonlinear steepest descent. We mention in this regard that RH problems with complicated contours that do not fit into the traditional framework arise in the analysis of initial-boundary value problems for integrable PDEs. For example, the analysis of the Degasperis–Procesi equation on the half-line leads to a RH problem with an unbounded jump contour involving nontransversal intersections, see Fig. 1.

## Appendix 1: Proof of proposition 3.1

We first prove a lemma.

### Lemma 6.1

Let $\Gamma \subset \mathbb {C}$ be an arc homeomorphic to *I* where *I* is either [0, 1], [0, 1), or (0, 1]. If $\gamma :I \rightarrow \Gamma$ is a homeomorphism, then $\Gamma$ is locally rectifiable if and only if $\gamma ((0,1))$ is locally rectifiable.

### Proof

We will prove that $\Gamma$ is rectifiable whenever $I = [0,1]$ and $\gamma ((0,1))$ is locally rectifiable; the other cases can easily be reduced to this case. Suppose $\gamma ((0,1))$ is locally rectifiable. Since $\gamma ((0,1))$ is contained in the bounded set $\gamma ([0,1])$, $\gamma ((0,1))$ is rectifiable. Let $a = t_0< t_1< \dots < t_N = b$ be a partition of a closed subinterval [*a*, *b*] of (0, 1). Since $\gamma ((0,1))$ is rectifiable,

$$\begin{aligned} \sup _{0< a< b< 1} \sup _{\begin{array}{c} \text {partitions} \\ \{t_i\}\text { of }[a,b] \end{array}} \sum _{j=1}^N |\gamma (t_j) - \gamma (t_{j-1})| < \infty . \end{aligned}$$

On the other hand, since $\gamma ([0,1])$ is compact,

$$\begin{aligned} \sup _{0< a< b< 1} (|\gamma (a) - \gamma (0)| + |\gamma (1) - \gamma (b)|) < \infty . \end{aligned}$$

Thus,

$$\begin{aligned} \sup _{0< a< b< 1} \sup _{\begin{array}{c} \text {partitions} \\ \{t_i\}\text { of }[a,b] \end{array}} \bigg (|\gamma (a) - \gamma (0)| + |\gamma (1) - \gamma (b)| + \sum _{j=1}^N |\gamma (t_j) - \gamma (t_{j-1})|\bigg ) < \infty , \end{aligned}$$

showing that $\Gamma$ is rectifiable. $\square$

We now prove Proposition 3.1. Let $\Gamma \subset \hat{\mathbb {C}}$ be a Carleson curve, that is, $\Gamma$ is connected and $\Gamma \cap \mathbb {C}$ is a locally rectifiable composed curve satisfying (2.1). We need to prove that $\psi (\Gamma ) \subset \hat{\mathbb {C}}$ is a Carleson curve. The proof is trivial if $c = 0$. Thus suppose $c \ne 0$. Since $\psi = \psi _4 \circ \psi _3 \circ \psi _2 \circ \psi _1$ is the composition of the four maps

$$\begin{aligned} \psi _1(z) = z + \frac{d}{c}, \quad \psi _2(z) = \frac{1}{z}, \quad \psi _3(z) = \frac{bc - ad}{c^2} z, \quad \psi _4(z) = z + \frac{a}{c}, \end{aligned}$$

and the operations of multiplication and translation by a complex number clearly preserve the family of Carleson curves, we may assume that $\psi (z) = z^{-1}$. Since $\psi (\Gamma ) \cap \mathbb {C}$ is Carleson if and only if each of its finite number of arcs is Carleson, we may assume that $\Gamma$ consists of a single (possibly unbounded) arc and that $\Gamma \subset \mathbb {C}$. Furthermore, Lemma 6.1 shows that we may discard any possible endpoints, and hence assume that $\Gamma$ is homeomorphic to the open interval (0, 1). Finally, if $0 \in \Gamma$, we may consider each of the two arcs that make up $\Gamma {\setminus } \{0\}$ separately. Thus, without loss of generality, let $\psi (z) = z^{-1}$ and let $\Gamma \subset \mathbb {C}$ be an arc homeomorphic to (0, 1) such that $0 \notin \Gamma$. Then $\psi (\Gamma )\subset \mathbb {C}$ is an arc homeomorphic to (0, 1) such that $0 \notin \psi (\Gamma )$. We need to prove that $\psi (\Gamma )$ is locally rectifiable and Carleson. Let $\gamma :(0,1) \rightarrow \Gamma$ be a homeomorphism.

### Lemma 6.2

If $\Gamma _0$ is a subarc of $\Gamma$ such that there exist constants $m, M \in (0, \infty )$ with the property that $m \le |z| \le M$ for all $z \in \Gamma _0$, then both $\Gamma _0$ and $\psi (\Gamma _0)$ are rectifiable and

6.1 $$\begin{aligned} m^2 |\psi (\Gamma _0)| \le |\Gamma _0| \le M^2 |\psi (\Gamma _0)|. \end{aligned}$$

### Proof

Since $\Gamma$ is locally rectifiable and $\Gamma _0$ is a bounded subarc, $\Gamma _0$ is rectifiable. Let $I \subset (0,1)$ be the subinterval of (0, 1) for which $\Gamma _0 = \gamma (I)$. Then *I* equals [*a*, *b*], [*a*, *b*), (*a*, *b*], or (*a*, *b*) for some $0< a< b < 1$. If $c \le t_0< t_1< \cdots < t_{N} \le d$ is a partition of a closed subinterval $[c,d] \subset I$, then

$$\begin{aligned} \sum _{j=1}^N |\gamma (t_j) - \gamma (t_{j-1})| = \sum _{j=1}^N |\psi (\gamma (t_j)) - \psi (\gamma (t_{j-1}))| |\gamma (t_j)| |\gamma (t_{j-1})| \end{aligned}$$

and so

$$\begin{aligned} m^2 \sum _{j=1}^N |\psi (\gamma (t_j)) - \psi (\gamma (t_{j-1}))|\le & {} \sum _{j=1}^N |\gamma (t_j) - \gamma (t_{j-1})|\nonumber \\\le & {} M^2 \sum _{j=1}^N |\psi (\gamma (t_j)) - \psi (\gamma (t_{j-1}))|. \end{aligned}$$

Taking the supremum over all partitions and all closed subintervals $[c,d] \subset I$, we find (6.1). $\square$

We next prove that $\psi (\Gamma )$ is locally rectifiable. Let [*a*, *b*] be a closed subinterval of (0, 1). Let $\Gamma _c = \gamma ([a,b])$. Since $\Gamma _c$ is compact and $0 \notin \Gamma _c$, $\Gamma _c$ is bounded and bounded away from 0; hence $\psi (\Gamma _c)$ is also bounded and bounded and bounded away from 0. It follows that $\psi (\Gamma _c)$ is rectifiable and Lemma 6.2 implies

6.2 $$\begin{aligned} |\psi (\Gamma _c) \cap D(0, r)|&= \sum _{n=1}^\infty \big |\psi (\Gamma _c) \cap \{2^{-n}r \le |w|< 2^{1-n}r\}\big | \nonumber \\&\le \sum _{n=1}^\infty r^2 2^{2 - 2n} \big |\Gamma _c \cap \{z|2^{n-1} r^{-1} < |z| \le 2^{n} r^{-1}\} \big |. \end{aligned}$$

By the Carleson property (2.2) of $\Gamma$ applied to $D(0, 2^{n} r^{-1})$, the right-hand side of (6.2) is bounded above by

$$\begin{aligned} C_\Gamma \sum _{n=1}^\infty r^2 2^{2 - 2n} 2^n r^{-1} = 4 C_\Gamma r, \end{aligned}$$

where $C_\Gamma > 0$ is a constant. Since the closed interval $[a,b] \subset (0,1)$ was arbitrary, it follows that

$$\begin{aligned} \sup _{0< a< b< 1} |\psi (\gamma ([a,b])) \cap D(0, r)| \le 4 C_\Gamma r < \infty \end{aligned}$$

for each $r>0$. This shows that $\psi (\Gamma )$ is locally rectifiable.

It remains to prove that $\psi (\Gamma )$ is Carleson. Let $w_0 \in \psi (\Gamma )$ and $r > 0$. Let $R = |w_0| + r$. Then

$$\begin{aligned} |\psi (\Gamma ) \cap D(w_0, r)|&= |\psi (\Gamma ) \cap D(w_0, r) \cap D(0, R) |\\&= \sum _{n=1}^\infty \big |\psi (\Gamma ) \cap D(w_0, r) \cap \{w| 2^{-n}R \le |w| < 2^{1-n} R\} \big |. \end{aligned}$$

In view of (6.1), this yields

where $z_0 = w_0^{-1}$. The set $\Gamma \cap \{z | |z^{-1} - z_0^{-1}|< r\} \cap \{z | 2^{n-1}R^{-1} < |z| \le 2^{n} R^{-1}\}$ is contained in the intersection of the two open disks $D(z_0, r |z_0| 2^{n} R^{-1})$ and $D(0, 2^{n+1} R^{-1})$. Hence we may use the Carleson property of $\Gamma$ on these disks to find

$$\begin{aligned} |\psi (\Gamma ) \cap D(w_0, r)|&\le C_\Gamma \sum _{n=1}^\infty 2^{2-2n}R^2 \min \big \{ r |z_0| 2^{n} R^{-1}, 2^{n+1} R^{-1}\big \} \\&\le 4 C_\Gamma R \min \{r |z_0|, 2\} = 4 C_\Gamma \min \{r(1 + r |w_0|^{-1}), 2(|w_0| + r)\}. \end{aligned}$$

where $C_\Gamma > 0$ is a constant. If $|w_0| \ge r$, then $4 C_\Gamma r(1 + r |w_0|^{-1}) \le 8 C_\Gamma r$. If $|w_0| < r$, then $8 C_\Gamma (|w_0| + r) < 16 C_\Gamma r$. Hence

$$\begin{aligned} |\psi (\Gamma ) \cap D(w_0, r)| < 16 C_\Gamma r, \end{aligned}$$

for all $w_0 \in \psi (\Gamma )$ and all $r > 0$. This proves that $\psi (\Gamma )$ is Carleson and completes the proof of Proposition 3.1. $\square$

## References

[1] Ablowitz MJ, Fokas AS (2003). Complex Variables: Introduction and Applications. Cambridge Texts in Applied Mathematics..

[2] Beals R, Coifman RR (1984). Scattering and inverse scattering for first order systems. Comm. Pure Appl. Math..

[3] Boutet de Monvel, A., Lenells, J., Shepelsky, D.: Long-time asymptotics for the Degasperis–Procesi equation on the half-line. Preprint arXiv:1508.04097

[4] Böttcher A, Karlovich YI (1997). Carleson Curves, Muckenhoupt Weights, and Toeplitz Operators, Progress in Mathematics, 154.

[5] Böttcher, A., Spitkovsky, I.M.: The factorization problem: some known results and open questions. Advances in harmonic analysis and operator theory, 101–122, Oper. Theory Adv. Appl. **229**, Birkhäuser/Springer, Basel, (2013)

[6] Clancey, K.F. , and Gohberg, I.: Factorization of matrix functions and singular integral operators. Operator Theory: Advances and Applications, vol. 3, pp. 234. Birkhäuser Verlag, Basel-Boston, Mass., (1981). ISBN:3-7643-1297-1

[7] David G (1984). Opérateurs intégraux singuliers sur certaines courbes du plan complexe. Ann. Sci. École Norm. Sup..

[8] Deift, P.: Orthogonal Polynomials and Random Matrices: A Riemann–Hilbert Approach, Courant Lecture Notes in Mathematics, 3, New York University, Courant Institute of Mathematical Sciences, New York. American Mathematical Society, Providence, RI (1999)

[9] Deift P, Venakides S, Zhou X (1997). New results in small dispersion KdV by an extension of the steepest descent method for Riemann–Hilbert problems. Int. Math. Res. Not..

[10] Deift P, Zhou X (1993). A steepest descent method for oscillatory Riemann–Hilbert problems. Asymptotics for the MKdV equation. Ann. Math..

[11] Deift P, Zhou X (2002). Perturbation theory for infinite-dimensional integrable systems on the line. A case study. Acta Math..

[12] Deift P, Zhou X (2002). A priori $$L^p$$-estimates for solutions of Riemann–Hilbert problems. Int. Math. Res. Not..

[13] Deift P, Zhou X (2003). Long-time asymptotics for solutions of the NLS equation with initial data in a weighted Sobolev space. Comm. Pure Appl. Math..

[14] Duren, P.L.: Theory of $$H^p$$ Spaces, Pure and Applied Mathematics, vol. 38, pp. 258. Academic Press, New York-London (1970)

[15] Fokas AS, Its AR (1996). The linearization of the initial-boundary value problem of the nonlinear Schrödinger equation. SIAM J. Math. Anal..

[16] Fokas AS, Its AR, Kapaev AA, Novokshenov VY (2006). Painlevé Transcendents. The Riemann–Hilbert Approach. Mathematical Surveys and Monographs.

[17] Gaier D (1987). Lectures on Complex Approximation.

[18] Gohberg, I., Kaashoek, M. A., Spitkovsky, I. M.: An overview of matrix factorization theory and operator applications, Factorization and integrable systems (Faro, 2000), 1–102, Oper. Theory Adv. Appl., 141, Birkhäuser, Basel, (2003)

[19] Its, A.R.: Asymptotic behavior of the solutions to the nonlinear Schrödinger equation, and isomonodromic deformations of systems of linear differential equations, Dokl. Akad. Nauk SSSR 261: 14–18 (in Russian). Soviet Math. Dokl. **24**(1982), 452–456 (1981). (in English)

[20] Kamvissis, S.: From stationary phase to steepest descent. Integrable systems and random matrices, 145–162, Contemp. Math., 458, Amer. Math. Soc., Providence, RI, (2008)

[21] Kamvissis S, Teschl G (2012). Long-time asymptotics of the periodic Toda lattice under short-range perturbations. J. Math. Phys..

[22] Khuskivadze G, Kokilashvili V, Paatashvili V (1998). Boundary value problems for analytic and harmonic functions in domains with nonsmooth boundaries. Applications to conformal mappings. Mem. Diff. Equ. Math. Phys..

[23] Lenells J (2013). The Degasperis–Procesi equation on the half-line. Nonlinear Anal..

[24] Litvinchuk GS, Spitkovskii IM (1987). Factorization of Measurable Matrix Functions, Operator Theory: Advances and Applications 25.

[25] Manakov, S.V.: Fraunhofer, Nonlinear, diffraction, Zh. Eksp. Teor. Fiz. 65, 1392–1398 (in Russian). Sov. Phys. JETP **38**(1974), 693–696 (1973). (in English)

[26] Muskhelishvili NI (1992). Singular Integral Equations. Boundary Problems of Function Theory and Their Application to Mathematical Physics.

[27] Priwalow II (1956). Randeigenschaften Analytischer Funktionen, Hochschulbücher für Mathematik.

[28] Rodin YL (1988). The Riemann Boundary Problem on Riemann Surfaces Mathematics and its Applications (Soviet Series).

[29] Zhou X (1989). The Riemann–Hilbert problem and inverse scattering. SIAM J. Math. Anal..

[30] Zverovič ÈI (1971). Boundary value problems in the theory of analytic functions in Hölder classes on Riemann surfaces. Russ. Math. Surv..

